# Long term health outcomes in people with diabetes 12 months after hospitalisation with COVID-19 in the UK: a prospective cohort study

**DOI:** 10.1016/j.eclinm.2024.103005

**Published:** 2024-12-27

**Authors:** Safoora Gharibzadeh, Ash Routen, Cameron Razieh, Francesco Zaccardi, Claire Lawson, Clare Gillies, Simon Heller, Melanie Davies, Helen Atkins, Stephen C. Bain, Nazir L. Lone, Krisnah Poinasamy, Tunde Peto, Elizabeth Robertson, Bob Young, Desmond Johnston, Jennifer Quint, Jonathan Valabhji, Khalida Ismail, Michael Marks, Alex Horsley, Annemarie Docherty, Ewen Harrison, James Chalmers, Ling-Pei Ho, Betty Raman, Chris Brightling, Omer Elneima, Rachel Evans, Neil Greening, Victoria C. Harris, Linzy Houchen-Wolloff, Marco Sereno, Aarti Shikotra, Amisha Singapuri, Louise Wain, Claudia Langenberg, John Dennis, John Petrie, Naveed Sattar, Olivia Leavy, Mattew Richardson, Ruth M. Saunders, Anne McArdle, Hamish McASuley, Tom Yates, Kamlesh Khunti, PHOSP-COVID Study Collaborative Group, C.E. Brightling, R.A. Evans, L.V. Wain, J.D. Chalmers, V.C. Harris, L.P. Ho, A. Horsley, M. Marks, K. Poinasamy, B. Raman, A. Shikotra, A. Singapuri, C.E. Brightling, R.A. Evans, L.V. Wain, R. Dowling, C. Edwardson, O. Elneima, S. Finney, N.J. Greening, B. Hargadon, V.C. Harris, L. Houchen--Wolloff, O.C. Leavy, H.J.C. McAuley, C. Overton, T. Plekhanova, R.M. Saunders, M. Sereno, A. Singapuri, A. Shikotra, C. Taylor, S. Terry, C. Tong, B. Zhao, D. Lomas, E. Sapey, C. Berry, C.E. Bolton, N. Brunskill, E.R. Chilvers, R. Djukanovic, Y. Ellis, D. Forton, N. French, J. George, N.A. Hanley, N. Hart, L. McGarvey, N. Maskell, H. McShane, M. Parkes, D. Peckham, P. Pfeffer, A. Sayer, A. Sheikh, A.A.R. Thompson, N. Williams, C.E. Brightling, W. Greenhalf, M.G. Semple, M. Ashworth, H.E. Hardwick, L. Lavelle-Langham, W. Reynolds, M. Sereno, R.M. Saunders, A. Singapuri, V. Shaw, A. Shikotra, B. Venson, L.V. Wain, A.B. Docherty, E.M. Harrison, A. Sheikh, J.K. Baillie, C.E. Brightling, L. Daines, R. Free, R.A. Evans, S. Kerr, O.C. Leavy, N.I. Lone, D. Lozano-Rojas, H.J.C. McAuley, K. Ntotsis, R. Pius, J. Quint, M. Richardson, M. Sereno, M. Thorpe, L.V. Wain, M. Halling-Brown, F. Gleeson, J. Jacob, S. Neubauer, B. Raman, S. Siddiqui, J.M. Wild, S. Aslani, G. Baxter, M. Beggs, C. Bloomfield, M.P. Cassar, A. Chiribiri, E. Cox, D.J. Cuthbertson, M. Halling-Brown, V.M. Ferreira, L. Finnigan, S. Francis, P. Jezzard, G.J. Kemp, H. Lamlum, E. Lukaschuk, C. Manisty, G.P. McCann, C. McCracken, K. McGlynn, R. Menke, C.A. Miller, A.J. Moss, T.E. Nichols, C. Nikolaidou, C. O'Brien, G. Ogbole, B. Rangelov, D.P. O'Regan, A. Pakzad, S. Piechnik, S. Plein, I. Propescu, A.A. Samat, L. Saunders, Z.B. Sanders, R. Steeds, T. Treibel, E.M. Tunnicliffe, M. Webster, J. Willoughby, J. Weir McCall, C. Xie, M. Xu, L.V. Wain, J.K. Baillie, H. Baxendale, C.E. Brightling, M. Brown, J.D. Chalmers, R.A. Evans, B. Gooptu, W. Greenhalf, H.E. Hardwick, R.G. Jenkins, D. Jones, I. Koychev, C. Langenberg, A. Lawrie, P.L. Molyneaux, A. Shikotra, J. Pearl, M. Ralser, N. Sattar, R.M. Saunders, J.T. Scott, T. Shaw, D. Thomas, D. Wilkinson, L.G. Heaney, A. De Soyza, D. Adeloye, C.E. Brightling, J.S. Brown, J. Busby, J.D. Chalmers, C. Echevarria, L. Daines, O. Elneima, R.A. Evans, J. Hurst, P. Novotny, C. Nicolaou, P. Pfeffer, K. Poinasamy, J. Quint, I. Rudan, E. Sapey, M. Shankar-Hari, A. Sheikh, S. Siddiqui, S. Walker, B. Zheng, J.R. Geddes, M. Hotopf, K. Abel, R. Ahmed, L. Allan, C. Armour, D. Baguley, D. Baldwin, C. Ballard, K. Bhui, G. Breen, K. Breeze, M. Broome, T. Brugha, E. Bullmore, D. Burn, F. Callard, J. Cavanagh, T. Chalder, D. Clark, A. David, B. Deakin, H. Dobson, B. Elliott, J. Evans, R.A. Evans, R. Francis, E. Guthrie, P. Harrison, M. Henderson, A. Hosseini, N. Huneke, M. Husain, T. Jackson, I. Jones, T. Kabir, P. Kitterick, A. Korszun, I. Koychev, J. Kwan, A. Lingford-Hughes, P. Mansoori, H. McAllister-Williams, K. McIvor, B. Michael, L. Milligan, R. Morriss, E. Mukaetova-Ladinska, K. Munro, A. Nevado-Holgado, T. Nicholson, C. Nicolaou, S. Paddick, C. Pariante, J. Pimm, K. Saunders, M. Sharpe, G. Simons, J.P. Taylor, R. Upthegrove, S. Wessely, G.P. McCann, S. Amoils, C. Antoniades, A. Banerjee, A. Bularga, C. Berry, P. Chowienczyk, J.P. Greenwood, A.D. Hughes, K. Khunti, C. Lawson, N.L. Mills, A.J. Moss, S. Neubauer, B. Raman, A.N. Sattar, C.L. Sudlow, M. Toshner, P.J.M. Openshaw, D. Altmann, J.K. Baillie, R. Batterham, H. Baxendale, N. Bishop, C.E. Brightling, P.C. Calder, C.M. Efstathiou, R.A. Evans, J.L. Heeney, T. Hussell, P. Klenerman, F. Liew, J.M. Lord, P. Moss, S.L. Rowland-Jones, W. Schwaeble, M.G. Semple, R.S. Thwaites, L. Turtle, L.V. Wain, S. Walmsley, D. Wraith, M.J. Rowland, A. Rostron, J.K. Baillie, B. Connolly, A.B. Docherty, N.I. Lone, D.F. McAuley, D. Parekh, A. Rostron, J. Simpson, C. Summers, R.G. Jenkins, J. Porter, R.J. Allen, R. Aul, J.K. Baillie, S. Barratt, P. Beirne, J. Blaikley, R.C. Chambers, N. Chaudhuri, C. Coleman, E. Denneny, L. Fabbri, P.M. George, M. Gibbons, F. Gleeson, B. Gooptu, B. Guillen Guio, I. Hall, N.A. Hanley, L.P. Ho, E. Hufton, J. Jacob, I. Jarrold, G. Jenkins, S. Johnson, M.G. Jones, S. Jones, F. Khan, P. Mehta, J. Mitchell, P.L. Molyneaux, J.E. Pearl, K. Piper Hanley, K. Poinasamy, J. Quint, D. Parekh, P. Rivera-Ortega, L.C. Saunders, M.G. Semple, J. Simpson, D. Smith, M. Spears, L.G. Spencer, S. Stanel, I. Stewart, A.A.R. Thompson, D. Thickett, R. Thwaites, L.V. Wain, S. Walker, S. Walsh, J.M. Wild, D.G. Wootton, L. Wright, S. Heller, M.J. Davies, H. Atkins, S. Bain, J. Dennis, K. Ismail, D. Johnston, P. Kar, K. Khunti, C. Langenberg, P. McArdle, A. McGovern, T. Peto, J. Petrie, E. Robertson, N. Sattar, K. Shah, J. Valabhji, B. Young, L.S. Howard, Mark Toshner, C. Berry, P. Chowienczyk, A. Lawrie, O.C. Leavy, J. Mitchell, J. Newman, L. Price, J. Quint, A. Reddy, J. Rossdale, N. Sattar, C. Sudlow, A.A.R. Thompson, J.M. Wild, M. Wilkins, S.J. Singh, W.D.-C. Man, J.M. Lord, N.J. Greening, T. Chalder, J.T. Scott, N. Armstrong, E. Baldry, M. Baldwin, N. Basu, M. Beadsworth, L. Bishop, C.E. Bolton, A. Briggs, M. Buch, G. Carson, J. Cavanagh, H. Chinoy, C. Dawson, E. Daynes, S. Defres, R.A. Evans, L. Gardiner, P. Greenhaff, S. Greenwood, M. Harvie, L. HOuchen-Wolloff, M. Husain, S. MacDonald, A. McArdle, H.J.C. McAuley, A. McMahon, M. McNarry, G. Mills, C. Nolan, K. O'Donnell, D. Parekh, Pimm, J. Sargent, L. Sigfrid, M. Steiner, D. Stensel, A.L. Tan, I. Vogiatzis, J. Whitney, D. Wilkinson, D. Wilson, M. Witham, D.G. Wootton, T. Yates, D. Thomas, N. Brunskill, S. Francis, S. Greenwood, C. Laing, K. Bramham, P. Chowdhury, A. Frankel, L. Lightstone, S. McAdoo, K. McCafferty, M. Ostermann, N. Selby, C. Sharpe, M. Willicombe, L. Houchen-Wolloff, J. Bunker, R. Gill, C. Hastie, R. Nathu, N. Rogers, N. Smith, A. Shaw, L. Armstrong, B. Hairsine, H. Henson, C. Kurasz, L. Shenton, S. Fairbairn, A. Dell, N. Hawkings, J. Haworth, M. Hoare, A. Lucey, V. Lewis, G. Mallison, H. Nassa, C. Pennington, A. Price, C. Price, A. Storrie, G. Willis, S. Young, P. Pfeffer, K. Chong-James, C. David, W.Y. James, C. Manisty, A. Martineau, O. Zongo, A. Sanderson, L.G. Heaney, C. Armour, V. Brown, T. Craig, S. Drain, B. King, N. Magee, D. McAulay, E. Major, L. McGarvey, J. McGinness, R. Stone, A. Haggar, A. Bolger, F. Davies, J. Lewis, A. Lloyd, R. Manley, E. McIvor, D. Menzies, K. Roberts, W. Saxon, D. Southern, C. Subbe, V. Whitehead, H. El-Taweel, J. Dawson, L. Robinson, D. Saralaya, L. Brear, K. Regan, K. Storton, J. Fuld, A. Bermperi, I. Cruz, K. Dempsey, A. Elmer, H. Jones, S. Jose, S. Marciniak, M. Parkes, C. Ribeiro, J. Taylor, M. Toshner, L. Watson, J. Weir McCall, J. Worsley, R. Sabit, L. Broad, A. Buttress, T. Evans, M. Haynes, L. Jones, L. Knibbs, A. McQueen, C. Oliver, K. Paradowski, J. Williams, E. Harris, C. Sampson, C. Lynch, E. Davies, C. Evenden, A. Hancock, K. Hancock, M. Rees, L. Roche, N. Stroud, T. Thomas-Woods, M. Babores, J. Bradley-Potts, M. Holland, N. Keenan, S. Shashaa, H. Wassall, E. Beranova, H. Weston, T. Cosier, L. Austin, J. Deery, T. Hazelton, C. Price, H. Ramos, R. Solly, S. Turney, L. Pearce, W. McCormick, S. Pugmire, W. Stoker, A. Wilson, N. Hart, L.A. Aguilar Jimenez, G. Arbane, S. Betts, K. Bisnauthsing, A. Dewar, P. Chowdhury, A. Chiribiri, A. Dewar, G. Kaltsakas, H. Kerslake, M.M. Magtoto, P. Marino, L.M. Martinez, C. O'Brien, M. Ostermann, J. Rossdale, T.S. Solano, E. Wynn, N. Williams, W. Storrar, M. Alvarez Corral, A. Arias, E. Bevan, D. Griffin, J. Martin, J. Owen, S. Payne, A. Prabhu, A. Reed, C. Wrey Brown, C. Lawson, T. Burdett, J. Featherstone, A. Layton, C. Mills, L. Stephenson, N. Easom, P. Atkin, K. Brindle, M.G. Crooks, K. Drury, R. Flockton, L. Holdsworth, A. Richards, D.L. Sykes, S. Thackray-Nocera, C. Wright, K.E. Lewis, A. Mohamed, G. Ross, S. Coetzee, K. Davies, R. Hughes, R. Loosley, L. O'Brien, Z. Omar, H. McGuinness, E. Perkins, J. Phipps, A. Taylor, H. Tench, R. Wolf-Roberts, L.S. Howard, O. Kon, D.C. Thomas, S. Anifowose, L. Burden, E. Calvelo, B. Card, C. Carr, E.R. Chilvers, D. Copeland, P. Cullinan, P. Daly, C.M. Efstathiou, L. Evison, T. Fayzan, H. Gordon, S. Haq, R.G. Jenkins, C. King, F. Liew, K. March, M. Mariveles, L. McLeavey, N. Mohamed, S. Moriera, U. Munawar, J. Nunag, U. Nwanguma, L. Orriss-Dib, D.P. O'Regan, A. Ross, M. Roy, E. Russell, K. Samuel, J. Schronce, N. Simpson, L. Tarusan, C. Wood, N. Yasmin, R. Reddy, A.-M. Guerdette, M. Hewitt, K. Warwick, S. White, A.M. Shah, C.J. Jolley, O. Adeyemi, R. Adrego, H. Assefa-Kebede, J. Breeze, M. Brown, S. Byrne, T. Chalder, A. Chiribiri, P. Dulawan, N. Hart, A. Hayday, A. Hoare, A. Knighton, M. Malim, C. O'Brien, S. Patale, I. Peralta, N. Powell, A. Ramos, K. Shevket, F. Speranza, A. Te, P. Beirne, A. Ashworth, J. Clarke, C. Coupland, M. Dalton, E. Wade, C. Favager, J. Greenwood, J. Glossop, L. Hall, T. Hardy, A. Humphries, J. Murira, D. Peckham, S. Plein, J. Rangeley, G. Saalmink, A.L. Tan, B. Whittam, N. Window, J. Woods, G. Coakley, D.G. Wootton, L. Turtle, L. Allerton, M. Beadsworth, A. Berridge, J. Brown, S. Cooper, A. Cross, D.J. Cuthbertson, S. Defres, S.L. Dobson, J. Earley, N. French, W. Greenhalf, H.E. Hardwick, K. Hainey, J. Hawkes, V. Highett, S. Kaprowska, G.J. Kemp, A.L. Key, S. Koprowska, L. Lavelle-Langham, N. Lewis-Burke, G. Madzamba, F. Malein, S. Marsh, C. Mears, L. Melling, M.J. Noonan, L. Poll, J. Pratt, E. Richardson, A. Rowe, M.G. Semple, V. Shaw, K.A. Tripp, B. Vinson, L.O. Wajero, S.A. Williams-Howard, J. Wyles, S.N. Diwanji, P. Papineni, S. Gurram, S. Quaid, G.F. Tiongson, E. Watson, B. Al-Sheklly, A. Horsley, C. Avram, P. Barran, J. Blaikely, M. Buch, N. Choudhury, D. Faluyi, T. Felton, T. Gorsuch, N.A. Hanley, T. Hussell, Z. Kausar, C.A. Miller, N. Odell, R. Osbourne, K. Piper Hanley, K. Radhakrishnan, S. Stockdale, D. Trivedi, A. De Soyza, C. Echevarria, A. Ayoub, J. Brown, G. Burns, G. Davies, H. Fisher, C. Francis, A. Greenhalgh, P. Hogarth, J. Hughes, K. Jiwa, G. Jones, G. MacGowan, D. Price, A. Sayer, J. Simpson, H. Tedd, S. Thomas, S. West, M. Witham, S. Wright, A. Young, M.J. McMahon, P. Neill, D. Anderson, H. Bayes, C. Berry, D. Grieve, I.B. McInnes, N. Basu, A. Brown, A. Dougherty, K. Fallon, L. Gilmour, K. Mangion, A. Morrow, K. Scott, R. Sykes, R. Touyz, E.K. Sage, F. Barrett, A. Donaldson, M. Patel, D. Bell, A. Brown, M. Brown, R. Hamil, K. Leitch, L. Macliver, J. Quigley, A. Smith, B. Welsh, G. Choudhury, J.K. Baillie, S. Clohisey, A. Deans, A.B. Docherty, J. Furniss, E.M. Harrison, S. Kelly, N.I. Lone, D.E. Newby, A. Sheikh, J.D. Chalmers, D. Connell, A. Elliott, C. Deas, J. George, S. Mohammed, J. Rowland, A.R. Solstice, D. Sutherland, C.J. Tee, N. Maskell, D. Arnold, S. Barrett, H. Adamali, A. Dipper, S. Dunn, A. Morley, L. Morrison, L. Stadon, S. Waterson, H. Welch, B. Jayaraman, T. Light, C.E. Bolton, P. Almeida, J. Bonnington, M. Chrystal, E. Cox, C. Dupont, S. Francis, P. Greenhaff, A. Gupta, L. Howard, W. Jang, S. Linford, L. Matthews, R. Needham, A. Nikolaidis, S. Prosper, K. Shaw, A.K. Thomas, L.P. Ho, N.M. Rahman, M. Ainsworth, A. Alamoudi, M. Beggs, A. Bates, A. Bloss, A. Burns, P. Carter, M. Cassar, K.M. Channon, J. Chen, F. Conneh, T. Dong, R.I. Evans, E. Fraser, X. Fu, J.R. Geddes, F. Gleeson, P. Harrison, M. Havinden-Williams, P. Jezzard, N. Kanellakis, I. Koychev, P. Kurupati, X. Li, E. Lukaschuk, K. McGlynn, H. McShane, C. Megson, K. Motohashi, S. Neubauer, D. Nicoll, G. Ogg, E. Pacpaco, M. Pavlides, Y. Peng, N. Petousi, J. Propescu, N. Rahman, B. Raman, M.J. Rowland, K. Saunders, M. Sharpe, N. Talbot, E. Tunnicliffe, W.D.-C. Man, B. Patel, R.E. Barker, D. Cristiano, N. Dormand, M. Gummadi, S. Kon, K. Liyanage, C.M. Nolan, S. Patel, O. Polgar, P. Shah, S.J. Singh, J.A. Walsh, J. Hurst, H. Jarvis, S. Mandal, S. Ahmad, S. Brill, L. Lim, D. Matila, O. Olaosebikan, C. Singh, M. Toshner, H. Baxendale, L. Garner, C. Johnson, J. Mackie, A. Michael, J. Pack, K. Paques, H. Parfrey, J. Parmar, N. Diar Bakerly, P. Dark, D. Evans, E. Hardy, A. Harvey, D. Holgate, S. Knight, N. Mairs, N. Majeed, L. McMorrow, J. Oxton, J. Pendlebury, C. Summersgill, R. Ugwuoke, S. Whittaker, W. Matimba-Mupaya, S. Strong-Sheldrake, S.L. Rowland-Jones, A.A.R. Thompson, J. Bagshaw, M. Begum, K. Birchall, R. Butcher, H. Carborn, F. Chan, K. Chapman, Y. Cheng, L. Chetham, C. Clark, Z. Coburn, J. Cole, M. Dixon, A. Fairman, J. Finnigan, L. Finnigan, H. Foot, D. Foote, A. Ford, R. Gregory, K. Harrington, L. Haslam, L. Hesselden, J. Hockridge, A. Holbourn, B. Holroyd-Hind, L. Holt, A. Howell, E. Hurditch, F. Ilyas, C. Jarman, A. Lawrie, E. Lee, J.-H. Lee, R. Lenagh, A. Lye, I. Macharia, M. Marshall, A. Mbuyisa, J. McNeill, S. Megson, J. Meiring, L. Milner, S. Misra, H. Newell, T. Newman, C. Norman, L. Nwafor, D. Pattenadk, M. Plowright, J. Porter, P. Ravencroft, C. Roddis, J. Rodger, P. Saunders, J. Sidebottom, J. Smith, L. Smith, N. Steele, G. Stephens, R. Stimpson, B. Thamu, N. Tinker, K. Turner, H. Turton, P. Wade, S. Walker, J. Watson, J.M. Wild, I. Wilson, A. Zawia, R. Aul, M. Ali, A. Dunleavy, D. Forton, N. Msimanga, M. Mencias, T. Samakomva, S. Siddique, J. Teixeira, V. Tavoukjian, J. Hutchinson, L. Allsop, K. Bennett, P. Buckley, M. Flynn, M. Gill, C. Goodwin, M. Greatorex, H. Gregory, C. Heeley, L. Holloway, M. Holmes, J. Kirk, W. Lovegrove, T.A. Sewell, S. Shelton, D. Sissons, K. Slack, S. Smith, D. Sowter, S. Turner, V. Whitworth, I. Wynter, L. Warburton, S. Painter, J. Tomlinson, C. Vickers, T. Wainwright, D. Redwood, J. Tilley, S. Palmer, G.A. Davies, L. Connor, A. Cook, T. Rees, F. Thaivalappil, C. Thomas, A. Butt, M. Coulding, H. Jones, S. Kilroy, J. McCormick, J. McIntosh, H. Savill, V. Turner, J. Vere, E. Fraile, J. Ugoji, S.S. Kon, H. Lota, G. Landers, M. Nasseri, S. Portukhay, A. Hormis, A. Daniels, J. Ingham, L. Zeidan, M. Chablani, L. Osborne, M. Marks, J.S. Brown, N. Ahwireng, B. Bang, D. Basire, R.C. Chambers, A. Checkley, R. Evans, M. Heightman, T. Hillman, J. Hurst, J. Jacob, S. Janes, R. Jastrub, M. Lipman, S. Logan, D. Lomas, M. Merida Morillas, A. Pakzad, H. Plant, J.C. Porter, K. Roy, E. Wall, B. Williams, M. Xu, D. Parekh, N. Ahmad Haider, C. Atkin, R. Baggott, M. Bates, A. Botkai, A. Casey, B. Cooper, J. Dasgin, K. Draxlbauer, N. Gautam, J. Hazeldine, T. Hiwot, S. Holden, K. Isaacs, T. Jackson, S. Johnson, V. Kamwa, D. Lewis, J.M. Lord, S. Madathil, C. McGhee, K. Mcgee, A. Neal, A. Newton Cox, J. Nyaboko, D. Parekh, Z. Peterkin, H. Qureshi, B. Rangelov, L. Ratcliffe, E. Sapey, J. Short, T. Soulsby, R. Steeds, J. Stockley, Z. Suleiman, T. Thompson, M. Ventura, S. Walder, C. Welch, D. Wilson, S. Yasmin, K.P. Yip, P. Beckett, C. Dickens, U. Nanda, C.E. Brightling, R.A. Evans, M. Aljaroof, N. Armstrong, H. Arnold, H. Aung, M. Bakali, M. Bakau, M. Baldwin, M. Bingham, M. Bourne, C. Bourne, N. Brunskill, P. Cairns, L. Carr, A. Charalambou, C. Christie, M.J. Davies, S. Diver, S. Edwards, C. Edwardson, O. Elneima, H. Evans, J. Finch, S. Glover, N. Goodman, B. Gootpu, N.J. Greening, K. Hadley, P. Haldar, B. Hargadon, V.C. Harris, L. Houchen-Wolloff, W. Ibrahim, L. Ingram, K. Khunti, A. Lea, D. Lee, D. Lozano-Rojas, G.P. McCann, H.J.C. McAuley, P. McCourt, T. Mcnally, G. Mills, A. Moss, W. Monteiro, K. Ntotsis, M. Pareek, S. Parker, A. Rowland, A. Prickett, I.N. Qureshi, R. Russell, N. Samani, M. Sereno, M. Sharma, A. Shikotra, S. Siddiqui, A. Singapuri, S.J. Singh, J. Skeemer, M. Soares, E. Stringer, T. Thornton, M. Tobin, E. Turner, L.V. Wain, T.J.C. Ward, F. Woodhead, J. Wormleighton, T. Yates, A. Yousuf, M.G. Jones, C. Childs, R. Djukanovic, S. Fletcher, M. Harvey, E. Marouzet, B. Marshall, R. Samuel, T. Sass, T. Wallis, H. Wheeler, R. Dharmagunawardena, E. Bright, P. Crisp, M. Stern, A. Wight, L. Bailey, A. Reddington, A. Ashish, J. Cooper, E. Robinson, A. Broadley, K. Howard, L. Barman, C. Brookes, K. Elliott, L. Griffiths, Z. Guy, D. Ionita, H. Redfearn, C. Sarginson, A. Turnbull, Y. Ellis, M. Marks, A. Briggs, K. Holmes, K. Poinasamy, S. Walker, M. Halling-Brown, G. Breen, M. Hotopf, K. Lewis, N. Williams

**Affiliations:** a Leicester Real World Evidence Unit, Diabetes Research Centre, Leicester General Hospital, University of Leicester, Leicester, LE5 4PW, UK; b Diabetes Research Centre, University of Leicester, Leicester General Hospital, Leicester, UK; c Health Analysis and Life Events Division, Office for National Statistics, Newport, UK; d Department of Cardiovascular Sciences, University of Leicester, Leicester, UK; e National Institute for Health Research (NIHR) Leicester Biomedical Research Centre, University Hospitals of Leicester NHS Trust and the University of Leicester, Leicester, UK; f NIHR Applied Research Collaboration–East Midlands, University of Leicester, Leicester, UK; g Department of Oncology and Metabolism, University of Sheffield, Sheffield, UK; h NIHR Leicester Biomedical Research Centre and Diabetes Research Centre, College of Life Sciences, University of Leicester, Leicester, UK; i University Hospitals of Leicester NHS Trust, Leicester, UK; j Swansea University Medical School, Swansea, UK; k Centre for Medical Informatics, The Usher Institute, University of Edinburgh, Edinburgh, UK; l Asthma and Lung UK, London, UK; m Department of Clinical Ophthalmology, Institute of Ophthalmology, University College London, London University, London, UK; n Diabetes UK, London, UK; o Faculty of Medicine, Imperial College London, London, UK; p NHLI, Imperial College London, London, UK; q Division of Metabolism, Digestion & Reproduction, Faculty of Medicine, Imperial College London, London, UK; r Department of Diabetes and Endocrinology, Chelsea and Westminster Hospital NHS Foundation Trust, London, UK; s Department of Clinical Research, London School of Hygiene & Tropical Medicine, London, UK; t Hospital for Tropical Diseases, University College London Hospital, London, UK; u Division of Infection, Immunity & Respiratory Medicine, Faculty of Biology, Medicine and Health, University of Manchester, Manchester, UK; v Manchester University NHS Foundation Trust, Manchester, UK; w University of Dundee, Ninewells Hospital and Medical School, Dundee, UK; x MRC Human Immunology Unit, University of Oxford, Oxford, UK; y Radcliffe Department of Medicine, University of Oxford, Oxford, UK; z Oxford University Hospitals NHS Foundation Trust, Oxford, UK; aa The Institute for Lung Health, NIHR Leicester Biomedical Research Centre, University of Leicester, Leicester, UK; ab Centre for Exercise and Rehabilitation Science, NIHR Leicester Biomedical Research Centre-Respiratory, University of Leicester, Leicester, UK; ac NIHR Leicester Biomedical Research Centre, University of Leicester, Leicester, UK; ad Department of Population Health Sciences, University of Leicester, Leicester, UK; ae MRC Epidemiology Unit, Institute of Metabolic Science, University of Cambridge, Cambridge, UK; af Precision Healthcare University Research Institute – PHURI, Queen Mary University London; And Berlin Institute of Health at Charité—Universitätsmedizin Berlin, Berlin, Germany; ag School of Medicine, Dentistry and Biomedical Sciences, Queen's University Belfast, Northern Ireland, UK; ah School of Health and Wellbeing, College of Medical, Veterinary and Life Sciences, University of Glasgow, Clarice Pears Building, Level 5 90 Byres Road, Glasgow, G12 8TB, UK; ai Robertson Centre for Biostatistics, School of Health and Wellbeing, College of Medical, Veterinary and Life Sciences, University of Glasgow, UK; aj Department of Musculoskeletal Biology, Institute of Ageing and Chronic Disease, University of Liverpool and MRC- Arthritis Research UK Centre for Integrated Research Into Musculoskeletal Ageing (CIMA), UK; ak Department of Diabetes and Endocrinology, Swansea Bay University Health Board, UK; al Institute of Psychiatry, Kings College London, London, UK; am NIHR Oxford Biomedical Research Centre, Oxford, UK

**Keywords:** Diabetes, Covid-19, Long Covid

## Abstract

**Background:**

People with diabetes are at increased risk of hospitalisation, morbidity, and mortality following SARS-CoV-2 infection. Long-term outcomes for people with diabetes previously hospitalised with COVID-19 are, however, unknown. This study aimed to determine the longer-term physical and mental health effects of COVID-19 in people with and without diabetes.

**Methods:**

The PHOSP-COVID study is a multicentre, long-term follow-up study of adults discharged from hospital between 1 February 2020 and 31 March 2021 in the UK following COVID-19, involving detailed assessment at 5 and 12 months after discharge. The association between diabetes status and outcomes were explored using multivariable linear and logistic regressions.

**Findings:**

People with diabetes who survived hospital admission with COVID-19 display worse physical outcomes compared to those without diabetes at 5- and 12-month follow-up. People with diabetes displayed higher fatigue (only at 5 months), frailty, lower physical performance, and health-related quality of life and poorer cognitive function. Differences in outcomes between diabetes status groups were largely consistent from 5 to 12-months. In regression models, differences at 5 and 12 months were attenuated after adjustment for BMI and presence of other long-term conditions.

**Interpretation:**

People with diabetes reported worse physical outcomes up to 12 months after hospital discharge with COVID-19 compared to those without diabetes. These data support the need to reduce inequalities in long-term physical and mental health effects of SARS-CoV-2 infection in people with diabetes.

**Funding:**

UK Research and Innovation and 10.13039/501100000272National Institute for Health Research. The study was approved by the Leeds West Research Ethics Committee (20/YH/0225) and is registered on the ISRCTN Registry (ISRCTN10980107).

**Research in context**

**Evidence before this study**

We searched PubMed for studies of the long-term effects of COVID-19 on individuals after hospitalisation, published from January 1 2020 to June 29 2023. We used the search terms (“COVID-19” and [“long-term” OR “sequelae” OR “consequences”] and “diabetes”) for Title only. We excluded studies that did not include diabetes populations and that were not in the English language. We identified one single-centre cross-sectional study of 112 hospitalised and non-hospitalised patients 9 months after acute infection. Fatigue, shortness of breath and chest pain were markedly higher in people with diabetes compared to those without. We also identified one prospective cohort study of 141 hospitalised patients 6 and 12 months after discharge. People with diabetes had significantly higher incidences of residual lung abnormalities at 6 months compared to those without. Finally, in a prospective cohort study of 248 COVID-19 patients discharged from hospital, diabetes status was associated with a greater risk of palpitations at 12 months. The impact of COVID-19 on long-term physical and mental health outcomes after hospitalisation in diabetes populations is not well understood.

**Added value of this study**

To our knowledge, we report on the first and largest prospective cohort study of the long-term physical and mental health effects of COVID-19 in people with diabetes one year after hospitalisation. Our findings show that people with diabetes who have survived hospital admission with COVID-19 display worse physical and mental health outcomes (fatigue (only at 5 months), frailty, number of symptoms, lower aerobic fitness, physical performance, cognitive function and health-related quality of life) compared to those without diabetes at 5- and 12-month follow-up. They also highlight that, differences in outcomes between diabetes status groups largely persist from 5 to 12-months.

**Implications of all the available evidence**

These data support the need to reduce inequalities in long-term physical and mental health effects of Long Covid in people with diabetes.

## Introduction

People living with diabetes have been at increased risk of severe outcomes and death during previous pandemics. For example, within hospitalised cohorts during the 2009 influenza A (H1N1) outbreak, people with diabetes had greater than four times increased odds of admission to an intensive care unit when compared to those without diabetes.^1^ Similarly, throughout the COVID-19 pandemic, people with diabetes have been at increased risk of hospitalisation, morbidity, and mortality following SARS-CoV-2 infection.^2^ A large population-level study which utilised data from over 60 million people in England compared COVID-19 outcomes in people with and without diabetes, adjusting analyses for age, sex, deprivation, ethnicity, and geographical region. This study reported a 3.5 fold and two-fold greater risk of COVID-19-related mortality in people with type 1 diabetes (T1DM) and type 2 diabetes (T2DM), respectively.^3^ Further, primary data analyses, and meta-analyses, have also reported increased severity of COVID-19 outcomes and mortality in people with diabetes.^2^^,^^4^^,^^5^ In cohorts of formerly hospitalised patients who have survived a serious illness, it is well evidenced that prolonged morbidity, including impaired functional status, poorer mental health, and greater healthcare service use can persist for several years.^6^ In addition, a large proportion (nearly 1 in 2) of people hospitalised or non-hospitalised with COVID-19 display a range of ongoing persistent symptoms (>12 weeks), which has been termed ‘Long Covid’.^7^ What is less clear, however, is the physical and mental health outcomes during longer-term follow-up for people with diabetes who were previously hospitalised with COVID-19. Only two previous small sample studies, including around 100 patients, have identified a number of long-term effects (at 6–9 months) in people with diabetes, compared to those without diabetes, including fatigue, shortness of breath, chest pain, and residual lung abnormalities.^8^^,^^9^ The Physical, cognitive, and mental health impacts of COVID-19 after hospitalisation (Post-Hospitalisation COVID-19 study, or PHOSP-COVID) study, a UK multicentre, prospective cohort, collected data at 5 and 12 months post-discharge, including detailed recording of symptoms and physiological and biochemical testing.^10^ From the initial 5-month follow-up of PHOSP-COVID data in 1077 discharged patients, only 29% of 830 participants reported feeling fully recovered, and 20% of 806 had a new disability. More than a quarter of the cohort had clinically significant symptoms of anxiety and depression and 12.2% reported symptoms of post-traumatic stress disorder (PTSD).^10^ In terms of physical functioning, 46.2% scored 10 or less on the short physical performance battery (SPPB), which indicates functional impairment.^10^ The proportion of individuals reporting full recovery was unchanged in a subsequent analyses of 12 month data from the same cohort (n = 807).^11^ In this cohort, the most common comorbidities were cardiovascular, respiratory, and type 2 diabetes (19.8%), with two or more comorbidities among factors associated with not recovering. There were also either no or minimal improvements in anxiety, depression, PTSD, physical function (SPPB and ISWT), and other outcomes.^11^ The longer term physical and mental health outcomes for people with diabetes who were previously hospitalised with COVID-19, however, remains to be explored, and there are currently no published data on this globally. The purpose of the present study therefore was to utilise the PHOSP-COVID cohort to examine the longer-term physical and mental health effects of SARS-CoV-2 infection in people with diabetes 12 months following hospital discharge and understand whether there are differences in COVID-19 recovery in those with and without diabetes.

## Methods

### Study design and participants

This analysis was conducted within the framework of PHOSP-COVID, a prospective cohort study in the UK. PHOSP-COVID is a multicentre, long-term follow-up study that recruited adults (≥18 years) who were discharged from one of the 83 National Health Service (NHS) hospitals across England, Northern Ireland, Scotland, and Wales following a diagnosis of COVID-19 (confirmed or clinically suspected) before March 31, 2021.^12^ COVID-19 status was ascertained by a reverse transcriptase polymerase chain reaction (RT-PCR) test for SARS-CoV-2 or a clinician diagnosis. Individuals were excluded if they attended the emergency department but were not admitted to hospital or if they had an existing condition with a life expectancy of less than 6 months.^13^ The study was approved by the Leeds West Research Ethics Committee (20/YH/0225) and is registered on the ISRCTN Registry (ISRCTN10980107). The recruitment process and study design have been reported previously.^10^

### Procedures

Participants were invited to attend two research visits at 5 (range 2–7) months and 12 (range 10–14) months post-discharge. from hospital.^14^ Participants were also allowed to attend a 1-year visit only if they were outside the time period for a 5-month visit at the time of consent and were discharged before November 30, 2020. At both visits, a core set of outcome variables were collected; the outcomes and how they were collected are listed in Tables S1 and S2. These variables included baseline demographics, and PCR test for SARS-CoV-2, as well as a range of physical and mental health measures. In addition to this follow-up data, acute admission details were captured.

The outcomes used in the present analysis include: Anxiety which was assessed using the Generalised Anxiety Disorder 7-item scale (GAD-7), with scores ranging from 0 to 21. A threshold of 8 was used, where a GAD score greater than 8 suggested at least mild-moderate anxiety,^15^ fatigue (Functional Assessment of Chronic Illness Therapy—Fatigue (FACIT) and Visual Analogue Scale (VAS), The Montreal Cognitive Assessment (MoCA), with a total score ranging from 0 to 30, total scores below 23 were indicative of at least mild cognitive impairment,^16^ physical performance/exercise capacity (incremental shuttle walk test (ISWT)), and health-related quality of life (EQ-5D-5L) (Table S1). Treatments and organ support received were obtained from hospital notes by the study team at each site. Diabetes status was determined retrospectively through hospital records completed by the treating physician. A recording of diagnosed diabetes or evidence of a pre-hospitalisation prescription of glucose lowering medication were used to define diabetes for the purposes of this study.^17^

### Role of funding

The funder of the study had no role in study design, data collection, data analysis, data interpretation, or writing of the report.

### Statistical analysis

Univariable normality of continuous variables were checked by Shapiro–Francia tests and graphical methods (such as box plot and Q–Q plot)^18^^,^^19^ and normally distributed variables were described by reporting the mean (standard deviation); median and inter-quartile ranges were used to report non-normally distributed variables Participants were categorised based on their diabetes status into two groups: “yes” (with diabetes) and “no” (without diabetes), without further differentiation between type 1 or type 2 diabetes. Subsequently, for each research visit, differences between diabetes groups in relation to continuous outcomes were assessed using either independent t-tests or Welch t-tests, depending on the homogeneity of variances. In instances of non-normality, the Mann–Whitney U test was applied. Wilcoxon signed rank test was applied to evaluate the change between 5 and 12 months in both groups.

Categorical outcomes, delineated by frequency and percent distribution in each group, were analysed using chi-squared tests. In cases where 20% of cells exhibited an expected frequency of less than 5, the Fisher's exact method was employed for comparison. Wilcoxon signed-rank tests were used to compare paired data within diabetes status groups between 5-month and 1-year visit.

Selection bias is one of the most common sources of bias in observational studies, particularly where there is attrition at follow-up. In order to reduce selection bias, Inverse Probability of Censoring Weighting (IPCW) was used.^20^ The probability of censoring was estimated using a logistic regression model and the inverse of these probabilities was used as the weight. Stabilised weights were applied as they are generally less variable than standard weights.^21^ Using disjunctive cause criterion,^22^ variables including age (with four knots, placed on the 5%, 35%, 65% and 95% quantiles following Harrell's suggestion),^23^ sex, diabetes status, educational level, ethnicity, deprivation index, hospitalisation duration, referred to another specialty, number of comorbidities, muscle ache were entered into the model with censoring as the outcome. A sandwich variance estimator with clustering by subject was used to obtain valid, but conservative, confidence intervals for IPCW estimators.^24^

The association between outcomes and diabetes status at both time points was evaluated using logistic regression or linear regression models, as applicable. Association of diabetes with outcomes of interest reported as beta coefficient (95% CI) or Odds Ratio (95% CI) for the binary outcome.

Initially, adjustments were made in a hierarchical manner. Model 1 was adjusted for age and sex, capturing their potential confounding effects. Model 2 was adjusted for age, sex, index of multiple deprivation, ethnicity, and education to account for broader sociodemographic factors. In Model 3, all covariates from Model 2 were included, with the addition of body mass index (BMI) considering its relevance to diabetes and the outcomes under investigation. Finally, Model 4 included the covariates from Model 3 and further incorporated the “number of comorbidities” as an additional factor. The number of comorbidities for patients with diabetes is defined as “plus one, “all of the patients with diabetes already have at least one co-morbidity.

To assess the association between the measurement taken at 5 months and the outcome observed at 1 year, an alternative strategy was employed.

In addition to the primary analyses, the severity of symptoms at baseline was further evaluated by including admission to the intensive care unit (ICU) as a variable in models 1–4.

The four aforementioned models (Models 1–4) were adjusted for the potential influence of the 5-month measurements. This adjustment aimed to account for any potential changes in the outcomes during the study period.

Fractional polynomials were employed to confirm the linearity assumption between the continuous variables and the outcome variable.^25^^,^^26^

All analyses were performed using Stata (16.0), and p < 0.05 were considered statistically significant.

## Results

**Fig. 1:**
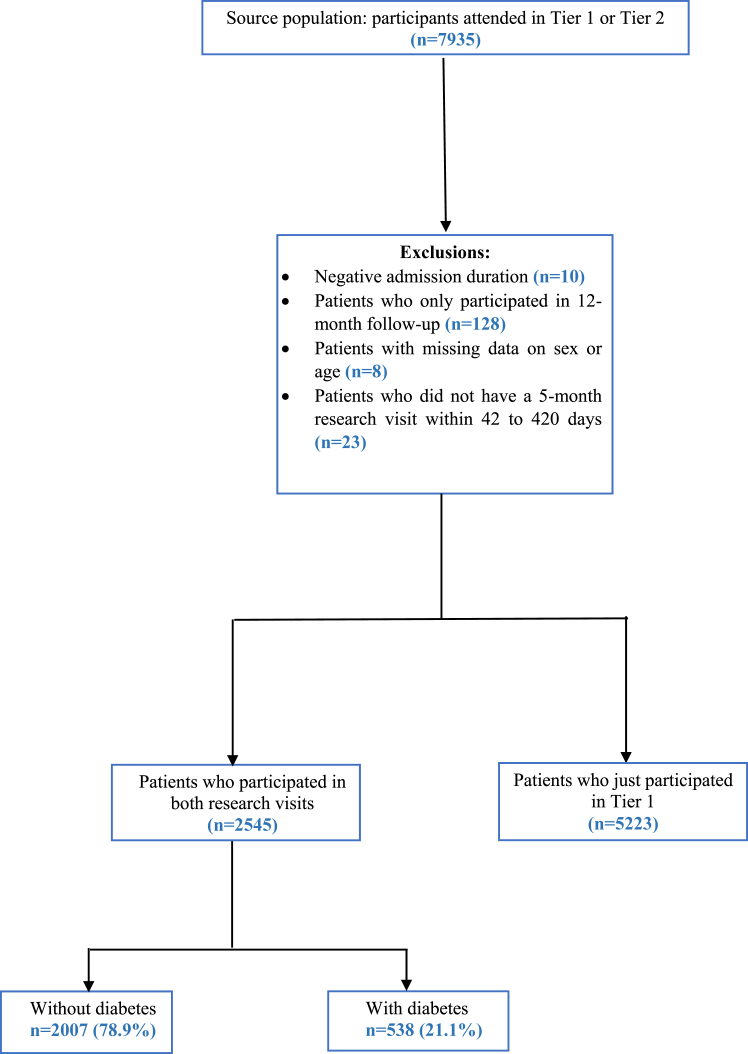
Flow diagram of study participants.

This analysis included a total of 2545 (38.8% females; 58 [±12.6] years, 74.8% White) patients discharged from hospital between 1 February 2020 and 31 March 2021 (Fig. 1). Of these, 538 (35.5% females; 61.1 [±11.3] years) had diabetes, with 65.3% being White, living with obesity (63.3%) and living with multiple long-term conditions (79.6% with ≥2 comorbidities). Comparatively, there were 2007 (39.7% females; 57.2 [±12.8] years) individuals living without diabetes; this group had proportionally more individuals who were White (77.4%), and fewer living with obesity (53.5%) and multiple long-term conditions (16.8%) than the diabetes group. 971 (38.1%) received the first dose of the vaccine, and 876 (34.4%) received both doses. The proportion of patients who received both doses was higher in patients with diabetes overall (37.3% vs. 33.6%, respectively, p < 0.0001). The full descriptive profile of the cohort, stratified by diabetes status, is displayed in Table 1, with further information on the cohort by tier of recruitment found in Table S3.

**Table 1:** Baseline characteristics of study participants.

	Total	Diabetes	No diabetes	p-value
	N = 2545	N = 538	N = 2007	
**Age at admission (years)**	58.0 (12.6)	61.1 (11.3)	57.2 (12.8)	<0.0001
**Sex at birth**				0.081
Female	988 (38.8%)	191 (35.5%)	797 (39.7%)	
Male	1557 (61.1%)	347 (64.5%)	1210 (60.2%)	
**Ethnicity**				
White	1893 (74.8%)	350 (65.3%)	1543 (77.4%)	<0.0001
South Asian	198 (7.8%)	58 (10.8%)	140 (7.0%)	
Black	180 (7.1%)	66 (12.3%)	114 (5.7%)	
Mixed/other	259 (10.2%)	62 (11.6%)	197 (9.9%)	
**Education level**				
None or primary	122 (5.4%)	35 (7.2%)	87 (4.9%)	0.00078
Secondary or college (NVQ 3–4)	1373 (60.3%)	318 (65.2%)	1055 (59.0%)	
Degree or higher	782 (34.3%)	135 (27.7%)	647 (36.2%)	
**Income (£)**				
<19 K	385 (21.2%)	97 (26.2%)	288 (19.9%)	0.0021
19–48 K	862 (47.4%)	181 (48.9%)	681 (47.0%)	
>48 K	573 (31.5%)	92 (24.9%)	481 (33.2%)	
**Index of multiple deprivation quintiles**				
1 (most deprived)	578 (23.1%)	146 (27.6%)	432 (21.9%)	<0.0001
2	584 (23.3%)	126 (23.8%)	458 (23.2%)	
3	435 (17.4%)	103 (19.5%)	332 (16.8%)	
4	436 (17.4%)	86 (16.3%)	350 (17.7%)	
5 (least deprived)	468 (18.7%)	67 (12.7%)	401 (20.3%)	
**Obesity (BMI >30 kg/m**^**2**^**)**	1001 (55.5%)	233 (63.3%)	768 (53.5%)	0.033
**Smoking status**				
Never	1436 (58.8%)	277 (53.5%)	1159 (60.2%)	0.0049
Ex-smoker	928 (38.0%)	228 (44.0%)	700 (36.3%)	
Smoker	80 (3.3%)	13 (2.5%)	67 (3.5%)	
**No. comorbidities**				
0	1194 (46.9%)	0 (0.0%)	1194 (59.5%)	<0.0001
1	586 (23.0%)	110 (20.4%)	476 (23.7%)	
2	407 (16.0%)	199 (37.0%)	208 (10.4%)	
≥2	358 (14.1%)	229 (42.6%)	129 (6.4%)	
Admission to ICU	825 (34.8)	201 (40.3)	624 (33.4)	<0.0001
**IHD**	172 (6.8%)	59 (11.0%)	113 (5.6%)	<0.0001
**MI**	108 (4.2%)	33 (6.1%)	75 (3.7%)	0.014
**HF**	41 (1.6%)	15 (2.8%)	26 (1.3%)	0.015
**AF/flutter**	118 (4.6%)	27 (5.0%)	91 (4.5%)	0.64
**Hypertension**	878 (34.5%)	325 (60.4%)	553 (27.6%)	<0.0001
**Congenital heart disease**	13 (0.5%)	4 (0.7%)	9 (0.4%)	0.39
**Valve disease**	40 (1.6%)	11 (2.0%)	29 (1.4%)	0.32
**Pacemaker/ICD**	21 (0.8%)	3 (0.6%)	18 (0.9%)	0.44
**Peripheral vascular disease**	36 (1.4%)	17 (3.2%)	19 (0.9%)	<0.0001
**Hypercholesterolemia**	500 (19.6%)	226 (42.0%)	274 (13.7%)	<0.0001
**Other cardiac condition**	56 (2.2%)	17 (3.2%)	39 (1.9%)	0.088
**CVA/TIA**	107 (4.2%)	40 (7.4%)	67 (3.3%)	<0.0001
**Chronic Kidney Disease**	107 (4.2%)	50 (9.3%)	57 (2.8%)	<0.0001
**Time from 1st symptom to admission (days)**	8.0 (6.0,11.0)	8.0 (5.0,11.0)	8.0 (6.0,11.0)	0.031
**Admission duration (days)**	8.0 (4.0, 16.0)	9.0 (5.0, 18.0)	7.0 (4.0, 15.0)	<0.0001
**No. of symptoms on admission**	5 (3,7)	5 (3,7)	5 (3,7)	0.11
**PCR positive test**	2164 (93.0%)	478 (95.8%)	1686 (92.3%)	<0.0001
**WHO clinical progression scale**				
Class 3/4 (no continuous O2)	392 (15.9%)	63 (12.0%)	329 (16.9%)	0.014
Class 5 (continuous O2)	1055 (42.7%)	221 (42.1%)	834 (42.9%)	
Class 7 (cpap or bpap or high flow O2)	584 (23.7%)	144 (27.4%)	440 (22.6%)	
Class 7–9 (imv or ecmo)	437 (17.7%)	97 (18.5%)	340 (17.5%)	
**Proning required**	469 (20.5%)	94 (19.3%)	375 (20.9%)	0.45
**Renal replacement therapy**	104 (4.3%)	30 (5.8%)	74 (3.8%)	0.049
**Pulmonary embolism**	242 (9.9%)	44 (8.5%)	198 (10.3%)	0.21
**Renal failure requiring haemodialysis**	82 (3.4%)	27 (5.2%)	55 (2.9%)	<0.0001
**Antibiotics**	1951 (78.6%)	423 (80.7%)	1528 (78.0%)	0.18
**Systemic steroids**	1387 (57.3%)	293 (57.2%)	1094 (57.3%)	0.97
**Anticoagulation**	1104 (45.5%)	219 (43.0%)	885 (46.2%)	0.21
**Lowest eGFR (ml/min/1.73 m**^**2**^**)**	80.5 (61.0, 91.0)	71.0 (50.0, 91.0)	83.0 (63.0, 91.0)	<0.0001
**eGFR <60 mL/min per 1.73 m**^**2**^	2234 (87.8%)	471 (87.5%)	1763 (87.8%)	0.85
**Alanine transaminase (U/L)**	60.0 (34.0, 109.0)	52.0 (32.0, 89.0)	63.0 (34.0, 113.0)	<0.0001
**Disability**	521 (20.7%)	142 (26.7%)	379 (19.1%)	<0.0001
**Feel fully recovered from COVID19**^a^	475 (18.8%)	104 (19.3%)	374 (18.6%)	0.92
**Refer to another speciality**	276 (10.8%)	64 (20.9%)	212 (18.3%)	0.29
**Muscle ache(yes)**	933 (44.05%)	176 (38.60%)	757 (45.55%)	<0.0001

Data are presented as mean (SD) or median (IQR) for continuous measures, and n (%) for categorical measures.

a After 12 months.

Table S8 shows the comparison between censored and followed participants at baseline in variables that were used for IPW. The percentage of people with diabetes, sex, and education were the same between the two groups, while the remaining factors differed between the two groups.

The median (interquartile range) of HbA1c (%) levels in people with diabetes at 5 months was 7.3 (6.5–8.0), and at 12 months after discharge, it was 7.2 (6.5–8.7). The change in HbA1c levels from 5 months to 1 year in people with diabetes was statistically significant (Wilcoxon signed-rank test's p-value = 0.021). The correlation between continuous outcomes and HbA1c levels was examined, and none of them were found to be statistically significant. Similarly, the distribution of HbA1c levels and binary outcomes one year after discharge was not statistically significant.

At 5- and 12-months after discharge, differences in anxiety, fatigue, cognitive impairment, physical performance/exercise capacity, frailty, and health-related quality of life between diabetes status groups are shown in Table 2. Fatigue VAS scores were higher at month 5, but not 12, in individuals with diabetes, and Fatigue FACIT scores were greater (with a lower score indicating greater burden) in people with diabetes at both timepoints.

**Table 2:** Patient-reported outcome measures, at 5 months and 1 year after hospital discharge, stratified by diabetes status.

	5-month				12-month			
	Total	Diabetes	No diabetes	p-value	Total	Diabetes	No diabetes	p-value
	N = 2545	N = 538	N = 2007		N = 1827	N = 396	N = 1431	
**GAD-7** >8	586 (25.4%)	129 (26.2%)	457 (25.2%)	0.67	364 (23.3%)	76 (22.8%)	288 (23.4%)	0.79
**Fatigue** FACIT	38.0 (24.9, 45.0)	36.0 (24.0–44.0)	39.0 (25.0, 46.0)	0.031	40.0 (28.0, 46.0)	37.0 (26.0, 45.0)	40.0 (28.0, 47.0)	<0.0001
**Fatigue** VAS**–**before (1–10 [worst])	0.0 (0.0, 2.0)	1.0 (0.0, 3.0)	0.0 (0.0, 2.0)	<0.0001	0.0 (0.0, 2.0)	0.0 (0.0, 2.0)	0.0 (0.0, 2.0)	0.16
**Fatigue** VAS–now	3.0 (0.0, 6.0)	4.0 (0.0, 7.0)	3.0 (0.0, 6.0)	0.033	3.0 (0.0, 6.0)	3.0 (0.0, 6.0)	2.0 (0.0, 5.0)	0.19
**Fatigue** VAS–change	2.0 (0.0, 4.0)	1.0 (0.0, 4.0)	2.0 (0.0, 4.0)	0.37	1.0 (0.0, 4.0)	2.0 (0.0, 5.0)	1.0 (0.0, 4.0)	0.88
**MOCA ≤23**	312 (15.2%)	100 (23.6%)	212 (13.0%)	<0.0001	130 (6.3%)	46 (10.9%)	84 (5.2%)	<0.0001
**ISWT distance (m)**	360.0 (230.0, 540.0)	290.0 (190.0, 440.0)	370.0 (250.0, 560.0)	<0.0001	380.0 (260.0, 570.0)	340.0 (210.0, 450.0)	420.0 (270.0, 590.0)	<0.0001
**ISWT % predicted**	64.9 (16.2)	64.8 (14.7)	64.9 (16.6)	0.93	64.3 (16.5)	63.2 (14.5)	64.5 (16.9)	0.27
**Rockwood frailty** score	2.7 (1.1)	3.2 (1.2)	2.6 (1.1)	<0.0001	2.7 (1.1)	3.1 (1.1)	2.6 (1.1)	<0.0001
**Rockwood frailty** level								
Very fit	289 (12.7%)	23 (4.8%)	266 (14.8%)	<0.0001	233 (14.2%)	24 (6.8%)	209 (16.3%)	<0.0001
Well or managing well	1497 (65.8%)	296 (62.3%)	1201 (66.7%)		1065 (64.9%)	225 (63.6%)	840 (65.3%)	
Vulnerable	358 (15.7%)	109 (22.9%)	249 (13.8%)		251 (15.3%)	77 (21.8%)	174 (13.5%)	
Mildly frail	78 (3.4%)	24 (5.1%)	54 (3.0%)		58 (3.5%)	14 (4.0%)	44 (3.4%)	
Moderate or higher frail severity	54 (2.4%)	23 (4.8%)	31 (1.7%)		33 (2.0%)	14 (4.0%)	19 (1.5%)	
**EQ-5D** before (1–100 [best])	85.0 (70.0, 90.0)	80.0 (64.0, 90.0)	85.0 (75.0, 90.0)	<0.0001	85.0 (70.0, 90.0)	80.0 (64.0, 90.0)	85.0 (75.0, 90.0)	<0.0001
**EQ-5D** now	75.0 (60.0, 85.0)	70.0 (50.0, 80.0)	75.0 (60.0, 85.0)	<0.0001	75.0 (60.0, 88.0)	70.0 (50.0, 80.0)	75.0 (60.0, 90.0)	<0.0001
**EQ-5D** change	−5.0 (−20.0, 0.0)	−5.0 (−16.0, 0.0)	−8.0 (−20.0, 0.0)	0.0012	−7.0 (−20.0, 0.0)	−5.0 (−20.0, 0.0)	−8.0 (−20.0, 0.0)	0.19
**EQ5D**-VAS	70.0 (50.0, 80.0)	65.0 (50.0, 80.0)	70.0 (50.0, 80.0)	<0.0001	70.0 (50.0, 80.0)	65.0 (50.0, 80.0)	70.0 (50.0, 80.0)	<0.0001

Data are presented as mean (SD) or median (IQR) for continuous measures, and n (%) for categorical measures.

Abbreviations: Fatigue (FACIT), fatigue (Functional Assessment of Chronic Illness Therapy; Fatigue VAS, Fatigue Visual Analogue Scale; EQ-5D-5L, EuroQol-5 Dimensions-5 Levels; ISWT distance, incremental; MoCA, The Montreal Cognitive Assessment; GAD-7, Generalised Anxiety Disorder 7-item scale.

The proportion of people with mild cognitive impairment (MoCA score <23) was greater in those with diabetes at both 5 and 12 months (5 months: 23.6% vs. 13.0%; 12 months: 10.9% vs. 5.2%). In addition, at both 5 and 12 months, individuals without diabetes achieved a greater distance incremental shuttle walk test.

Frailty remained consistent in those with and without diabetes from month 5 to month 12. Individuals living with diabetes reported elevated levels of frailty at both months 5 and 12 compared to those without diabetes (month 5: diabetes 3.2 (±1.2), no diabetes 2.6 (±1.1); month 12: diabetes 3.1 (±1.1), no diabetes 2.6 (±1.1)). In addition, for frailty classification, the proportion of individuals categorised as either ‘mildly frail’ or ‘moderately or higher frail severity’ was higher in people with diabetes than those without (moderately or higher frail severity at month 5: diabetes 4.8%, no diabetes 1.7%; month 12: diabetes 4.0%, no diabetes 1.5%) and decreased from month 5 to 12 in both groups. Quality of life also remained consistent from 5- to 12-months in those with and without diabetes, with quality-of-life scores being higher in those without diabetes at 5 and 12 months. The Wilcoxon signed rank test results showed that there were significant changes between the research visits in most areas, except for GAD scores and EQ-5D scores for both the total population and grouped patients (with and without DM) (Table S6). Another notable finding was that there wasn't a significant change in the Fatigue FACIT scale for people with DM between the 5 and 12-month intervals.

At 5 months, there was a negative association between diabetes status and the Fatigue FACIT score in Model 1 (−1.7, 95% CI: −3.0 to −0.3) and Model 2–1.7, 95% CI: −3.1 to −0.4). However, this association was not considerable after adjusting for additional covariates in subsequent models (Table 3). Diabetes status was positively associated with the Fatigue VAS-now score in all models, with Model 1 and Model 2 showing a significant effect (Table 3). Diabetes status had also a significant negative association with the health-related quality of life score in Model 1 to Model 3 (Table 3). Physical performance, measured by ISWT distance, was negatively associated with diabetes in the first three models (−91.4, −72.9, and −48.9 m, respectively), indicating that individuals with diabetes were able to walk shorter distances. However, this association became non-significant after adjusting for the number of comorbidities in Model 4. Diabetes was found to be associated with higher prevalence of “having mild cognitive impairment” (MoCA score <23) in Model 1, adjusted for age and sex (OR = 1.6, 95% CI: 1.2–2.2) (Table 4). This association persisted in Model 3, after adjustments for age, sex, deprivation, ethnicity, education, and BMI, with a odds ratio of 1.5 (95% CI: 1.0–2.1).

**Table 3:** Regression coefficient^a^ with 95% CI of continuous outcomes.

	5-month								12-Month							
	Model 1	p-value	Model 2	p-value	Model 3	p-value	Model 4	p-value	Model 1	p-value	Model 2	p-value	Model 3	p-value	Model 4	p-value
Fatigue FACIT	−1.70 (−3.04, −0.35)	0.013	−1.79 (−3.13, −0.44)	0.0091	−0.53 (−2.12, 1.05)	0.051	1.95 (−0.49, 4.39)	0.11	−2.49 (−4.10, −0.88)	0.0022	−2.50 (−4.11, −0.89)	0.0023	−1.48 (−3.40, 0.43)	0.13	0.35 (−2.52, 3.23)	0.89
Fatigue VAS	0.38 (0.01, 0.74)	0.041	0.36 (−0.00, 0.73)	0.052	0.20 (−0.24, 0.65)	0.36	−0.50 (−1.19, 0.19)	0.15	0.36 (−0.05, 0.78)	0.093	0.38 (−0.04, 0.80)	0.081	0.04 (−0.45, 0.55)	0.24	−0.42 (−1.14, 0.29)	0.24
EQ-5D now	−4.12 (−6.38, −1.85)	<0.0001	−4.16 (−6.42, −1.90)	<0.0001	−2.95 (−5.65, −0.24)	0.032	0.05 (−4.40, 4.50)	0.98	−4.81 (−7.54, −2.08)	<0.0001	−4.22 (−6.91, −1.54)	0.0021	−2.44 (−5.61, 0.73)	0.13	0.38 (−4.63, 5.39)	0.88
ISWT distance (m)	−91.48 (−117.37, −65.59)	<0.0001	−72.96 (−99.13, −46.62)	<0.0001	−48.94 (−80.55, −17.33)	<0.0001	23.99 (−19.88, 67.86)	0.28	−95.30 (−126.22, −64.38)	<0.0001	−79.94 (−111.50, −48.38)	<0.0001	−44.83 (−79.95, −9.70)	0.012	44.88 (−3.44, 93.21)	0.071

Abbreviations: Fatigue (FACIT), fatigue (Functional Assessment of Chronic Illness Therapy; Fatigue VAS: Fatigue Visual Analogue Scale; EQ-5D-5L, EuroQol-5 Dimensions-5 Levels; ISWT distance, incremental shuttle walk test distance.

Model 1: adjusted for age, sex; Model 2: adjusted for age, sex, index of multiple deprivation, Ethnicity, and education; Model 3: adjusted for Model2 + BMI; Model 4: Model 3 + number of long-term conditions.

a Comparing patients with diabetes vs. without diabetes.

**Table 4:** Odds Ratio (OR)^a^ (95% CI) binary outcomes.

	5-month								12-Month							
	Model 1	p-value	Model 2	p-value	Model 3	p-value	Model 4	p-value	Model 1	p-value	Model 2	p-value	Model 3	p-value	Model 4	p-value
MoCA score <23	1.68 (1.25, 2.24)	<0.0001	1.31 (0.95, 1.79)	0.095	1.51 (1.04, 2.18)	0.029	1.34 (0.75, 2.38)	0.31	2.01 (1.34, 3.02)	<0.0001	1.47 (0.96, 2.26)	0.075	1.36 (0.93, 2.24)	0.21	0.88 (0.42, 1.86)	0.75
Anxiety (GAD-7 score >8)	1.15 (0.89, 1.48)	0.26	1.10 (0.85, 1.43)	0.43	1.12 (0.82, 1.53)	0.45	0.89 (0.55, 1.42)	0.62	1.14 (0.83, 1.55)	0.38	1.01 (0.73, 1.40)	0.91	0.94 (0.63,1.41)	0.78	0.66 (0.36, 1.19)	0.17

Abbreviations: MoCA, The Montreal Cognitive Assessment; GAD-7, Generalised Anxiety Disorder 7-item scale.

Model 1: adjusted for age, sex; Model 2: adjusted for age, sex, index of multiple deprivation, Ethnicity, and education; Model 3: adjusted for Model 2 + BMI; Model 4: Model 3 + number of long-term conditions.

a Comparing patients with diabetes vs. without diabetes.

One year after hospitalisation, diabetes status was positively associated with a higher burden of fatigue, as indicated by lower scores on the Fatigue FACIT scale, in Model 1 and Model 2; however, this association became non-significant after adjusting for additional covariates in subsequent models (Table 3). Health-related quality of life (HRQOL) was negatively associated with diabetes status in the first two nested models: model 1, which adjusted for age and sex, showed an effect size of −4.8 (95% CI: −7.5 to −2.0) while Model 2, which included additional factors such as index of multiple deprivation, ethnicity, and education, showed an effect size of −4.2 (95% CI: −6.9 to −1.5). ISWT was also negatively associated with diabetes. Patients with diabetes reported lower physical performance by 101, 83, and 47 m in Model 1, Model 2, and Model 3, respectively, compared to patients without diabetes. Diabetes status was positively associated with mild cognitive impairment in model 1 at 12 months, but was not in Models 2–4; and anxiety was not associated with diabetes status in any of the models. Overall, both at 5 and 12 months, associations with all outcomes were attenuated when adjusting for BMI and the presence of other long-term conditions.

Table S7 shows the Odds Ratios (OR) with 95% confidence intervals for diabetes mellitus (DM) in all models adjusted for ICU admission at baseline. It was observed that the direction and significance of the effects remained consistent, although slight changes in the ORs were noted due to modifications in the model matrix.

## Discussion

To our knowledge, this is the first study to date to report on prospectively assessed physical and mental health effects of COVID-19 in people with diabetes. When compared to study recruits without diabetes, we found that people with diabetes displayed higher fatigue (only at 5 months), frailty, lower physical performance, and health-related quality of life and poorer cognitive function one year after hospitalisation. However, differences disappeared when accounting for body mass index and the number of comorbidities.

Fatigue scores showed improvement at 12 months, with no differences between diabetes status groups. Likewise, the proportion of individuals classified as either ‘mildly frail’ or ‘moderately or higher frail severity’ decreased from 5 to 12 months in people with diabetes, but not in those without. Differences in outcomes between diabetes status groups otherwise remained consistent from 5 to 12-months. The increased burden of a range of physical and mental health effects in people living with diabetes appears to remain, even 12 months following hospitalisation for COVID-19. This supports the need to reduce inequalities in long-term physical and mental health targeted at people living with diabetes following hospitalisation for COVID-19. However, some but not all of the differences were clinically meaningful. In Model 1 those with diabetes had an 89% (95% CI 1.3, 2.7%) higher risk of mild cognitive impairment and covered 95.3 (95% CI: 64.3, 126.2) fewer meters in the ISWT (physical performance/exercise capacity) which is clinically meaningful. However, differences in HRQOL and fatigue, although statistically significant, may not have been clinically meaningful.^27^, ^28^, ^29^, ^30^ Therefore, there is some evidence of meaningful inequalities in long-term physical and mental health targeted at people living with diabetes following hospitalisation for COVID-19.

In regression models we found that the association between diabetes status and some physical, cognitive, and mental health outcomes were predominantly improved when including body mass index and number of comorbidities, particularly at 5 months. The lack of significant associations may therefore be explained in part by the fact that at baseline the diabetes group had a greater proportion of obese individuals (63.3% vs. 53.5%), and greater number of comorbidities (>2 comorbidities: 42.6% vs. 6.4%). These variables have also been reported as risk factors for persistent symptoms following COVID-19 in non-hospitalised^31^ and hospitalised (comorbidities only) populations.^10^ Therefore, while having diabetes itself may negatively influence outcomes following COVID-19 hospitalisation, the poorer outcomes we identified for people living with diabetes may in part be due to obesity and pre-existing comorbidities.

Only two previous studies have examined the long-term physical and mental health effects of COVID-19 in diabetes populations. In a single-centre cross-sectional study of 112 hospitalised and non-hospitalised patients, Mechi et al^8^ collected symptom burden data via an interview administered survey at 9 months after acute infection. In comparison to the group of individuals without diabetes (n = 70), those with diabetes displayed markedly higher fatigue (76% vs. 53%, p = 0.01), shortness of breath (45% vs. 21%, p = 0.01), chest pain (31% vs. 13%, p = 0.02), and cough (26% vs. 13%, p = 0.07). The study was, however, single centre and cross-sectional, with a small sample.

A similar sized prospective cohort study conducted lung CT scans in 141 hospitalised patients 6- and 12-months following discharge. At 6 months people with diabetes (n = 52) and secondary hyperglycaemia (n = 48) had significantly higher incidences of residual lung abnormalities than non-diabetic controls (n = 41; 65.4% and 58.3%, respectively vs. 36.6%; p < 0.05).^9^ Numbers were too small to examine inferentially at 12-month follow-up. Again, these data are limited by the small sample in each patient sub-group and recruitment from only two hospitals. The strengths of our large, multicentre cohort study include the most comprehensive assessment of in-clinic and patient-reported outcomes in people living with diabetes previously hospitalised for COVID-19. However, this study has several limitations. There may be selection bias for individuals who returned for a 12 month follow-up visit.^11^ There is a higher proportion of men (∼60%) included within the cohort, and women have been reported to display worse long-term outcomes.^11^ However, the apparent paradox of more men being recruited when women are more commonly affected by Long Covid may be explained by the greater proportion of men who were hospitalised.^32^ Further limitations are the lack of a matched control group without SARS-CoV-2 infection, and the absence of pre-hospital patient data, as well as information on the proportion of patients who died in hospital. People with diabetes were at higher risk of dying in hospital from COVID-19, so our cohort could be considered a survivor cohort. It is therefore unclear if a greater proportion of people with diabetes, who would otherwise have experienced symptoms post-discharge, died pre-discharge. Furthermore, it's important to acknowledge the possibility of unmeasured confounding and residual confounding stemming from measurement errors in variables like the index of multiple deprivation. And in addition, there were only 26 patients with T1D, and so we were unable to conduct separate analysis for these patients. Similarly, we are unable to determine the distinct additional influence of Long Covid on outcomes beyond what would be anticipated solely from diabetes.

In conclusion, patients with diabetes who have survived hospital admission with COVID-19 display worse physical and mental health outcomes compared to those without diabetes at 5- and 12-month follow-up. Diabetes status was, however, not associated with key outcomes when accounting for some demographic and clinical variables. Taken together, with limited extant data, our findings support the need to provide intervention to improve inequalities in outcomes for people with diabetes with long-term health effects following SARS-CoV-2 infection.

## Contributors

KK conceptualised the study, SGh led the analysis. The manuscript was initially drafted by SGh and ACR, and further developed by CR, TY and KK. All authors contributed to data interpretation and critical review and revision of the manuscript. KK and TY had full access to all the data in the study and had final responsibility for the decision to submit for publication.

## Data sharing statement

The protocol, consent form, definition and derivation of clinical characteristics and outcomes, training materials, regulatory documents, requests for data access and other relevant study materials are available online at https://www.phosp.org.

## Declaration of interests

Dr. Gharibzadeh, Dr. Routen, Dr. Gillies, Dr. Harris, Dr. Lone, Dr. Dennis, Dr. Quint, Dr. McAuley, Mr. Sereno, Dr. Elneima, Dr. Saunders, Dr. Houchen-Wolloff, Dr. Razieh, Dr. Ho, Dr. Harrison, Dr. Raman, Dr. Parmar, Dr. Bain, Professor Langenberg, Dr. Peto, Dr. Petrie, Dr. Robertson, Dr. McArdle, Dr. Richardson, Mr. Poinasamy, Dr. Johnston, Ms. Atkins, and Dr Ismail: has nothing to disclose.

Dr. Greening: Reports grants from Wellcome Leap, Rejuvenate Biomed, and Eupnoos; grants and personal fees from GSK, Roche/Genentech; and personal fees from Chiesi, Pulmonx, and AstraZeneca, outside the submitted work.; Dr. Zaccardi: Reports personal fees from Menarini and grants and personal fees from Servier, outside the submitted work.; Dr. Yates: Reports grants from NIHR Leicester BRC, during the conduct of the study.; Dr. Chalmers: Reports grants and personal fees from AstraZeneca, Boehringer Ingelheim, Genentech, GlaxoSmithKline, Insmed, and Antabio; personal fees from Chiesi, Roche, Zambon, and Pfizer; grants from Grifols, outside the submitted work.; Dr. Singapuri: Reports grants from UKRI & NIHR (Joint funding) during the conduct of the study.; Dr. Docherty: Reports grants from the Wellcome Trust, during the conduct of the study.; Dr. Brightling: Reports grants from NIHR UKRI and Leicester NIHR BRC, during the conduct of the study; grants and personal fees from Areteia, AstraZeneca, Chiesi, Genentech, GlaxoSmithKline, Mologic, Novartis, Regeneron Pharmaceuticals, Roche, and Sanofi, outside the submitted work.; Dr. Lawson: Reports grants from NIHR, during the conduct of the study.; Dr. Marks: Reports grants from DHSC, during the conduct of the study.; Dr. Horsley: Reports grants from the National Institute of Health and Social Care Research (NIHR) Manchester Biomedical Research Centre, during the conduct of the study.; Dr. Wain: Reports grants from UKRI, NIHR, GSK/Asthma + Lung UK during the conduct of the study; grants from Roche, Orion Pharma, and the Wellcome Trust; grants and other support from GSK; other support from Boehringer Ingelheim and Galapagos; personal fees from the European Respiratory Society, outside the submitted work.; Dr. Davies: Reports grants, personal fees, and other support from Boehringer Ingelheim; personal fees and other support from Eli Lilly; grants, personal fees, and other support from Novo Nordisk; other support from Sanofi, Amgen, Biomea Fusion, Carmot, Roche, Zealand Pharma, and Regeneron; and grants, personal fees, and other support from AstraZeneca, outside the submitted work.; Dr. Sattar: Reports personal fees from Abbott Laboratories, AbbVie, Amgen, Hanmi Pharmaceuticals, Janssen, Menarini-Ricerche, Novo Nordisk, and Pfizer; grants and personal fees from AstraZeneca, Boehringer Ingelheim, Novartis, and Roche Diagnostics; personal fees from Sanofi, outside the submitted work.; Professor Jonathan Valabhji: Reports he is National Clinical Lead for Multiple Long-Term Conditions at NHS England and was National Clinical Director for Diabetes and Obesity at NHS England from April 2013 to September 2023.; Dr. Leavy: Reports grants from UKRI and NIHR, during the conduct of the study.; Dr. Heller: Reports other support from Vertex Pharma, Novo Nordisk, Zucara, and Eli Lilly, outside the submitted work.; Dr. Evans: Reports grants from UKRI/MRC/NIHR, personal fees from AstraZeneca/Evidera for Long COVID, and personal fees from Boehringer (June 2021) and Moderna (April 2023) during the conduct of the study; grants from the Wolfson Foundation; and non-financial support from ERS Group 01.02 Pulmonary Rehabilitation and Chronic Care Chair and the ATS Pulmonary Rehabilitation Assembly Chair, outside the submitted work.; Prof. Khunti reports: Payment or honoraria for lectures, presentations, speakers bureaus, manuscript writing or educational events, consulting Fees: Amgen, AstraZeneca, BMS, Boehringer Ingelheim, Lilly, Novo Nordisk, Sanofi, Servier, Pfizer, Roche, Daiichi-Sankyo, Embecta and Nestle Health Science and Grants: AstraZeneca, Boehringer Ingelheim, Lilly, MSD, Novo Nordisk, Roche, Sanofi, Servier, Oramed Pharmaceuticals, Roche, Daiichi-Sankyo, Applied Therapeutics. Bob Young, who has sadly passed away, was included with his family's consent.

## Appendix A Supplementary data

Supplemenary Material
