# Supplementary material for: Long term health outcomes in people with diabetes 12 months after hospitalisation with COVID-19 in the UK: a prospective cohort study
Source: eClinicalMedicine. 2024 Dec 27;79:103005. doi: 10.1016/j.eclinm.2024.103005 (PMC11743801; doi:10.1016/j.eclinm.2024.103005)
Supplement: Supplemenary Material [file mmc1.docx]

**Supplementary material**

**Long term health outcomes in people with Diabetes 12 Months After Hospitalisation with COVID-19 in the UK: A Prospective Cohort Study**

[PHOSP-COVID Collaborative Group 2](#_Toc182567914)

[Table S1- Outcome measures 12](#_Toc182567915)

[Table S2. Methods and thresholds for processing of variables and outcome measures 13](#_Toc182567916)

[Table S3 – PHOSP participants’ characteristics by tier 15](#_Toc182567917)

[Table S4- Linear Regression Models for Patient Reported Outcomes at 12-Months; Hierarchical Adjustment of 5-Month Measurements 16](#_Toc182567918)

[Table S5- Logistic Regression for Patient Reported Outcomes at 12-Months; Hierarchical Adjustment of 5-Month Measurements 16](#_Toc182567919)

[Table S6- Outcome change score from 5 to 12 months 17](#_Toc182567920)

[Table S7- Odds Ratio (with 95% confidence intervals) of diabetes in all four models, adjusted for admission to ITU at baseline. 17](#_Toc182567921)

[Table S8- comparing the baseline characteristics and variables adjusted for using inverse probability-of-censoring weighting) between censored and uncensored participants 18](#_Toc182567922)

[References: 19](#_Toc182567923)

# PHOSP-COVID Collaborative Group

**Core Management Group**

*Chief Investigator* C E Brightling, *Members* R A Evans (Lead Co-I), L V Wain (Lead Co-I), J D Chalmers, V C Harris, L P Ho, A Horsley, M Marks, K Poinasamy, B Raman, A Shikotra, A Singapuri

**PHOSP-COVID Study Central Coordinating Team**

C E Brightling (Chief Investigator), R A Evans (*Lead Co-I*), L V Wain (*Lead Co-I*), R Dowling, C Edwardson, O Elneima, S Finney, N J Greening, B Hargadon, V C Harris, L Houchen--Wolloff, O C Leavy, H J C McAuley, C Overton, T Plekhanova, R M Saunders, M Sereno, A Singapuri, A Shikotra, C Taylor, S Terry, C Tong, B Zhao

**Steering Committee**

*Co-chairs* D Lomas, E Sapey*, Institution representatives* C Berry, C E Bolton, N Brunskill, E R Chilvers, R Djukanovic, Y Ellis, D Forton, N French, J George, N A Hanley, N Hart, L McGarvey, N Maskell, H McShane, M Parkes, D Peckham, P Pfeffer, A Sayer, A Sheikh, A A R Thompson, N Williams and core management group representation

**Executive Board**

*Chair* C E Brightling, representation from the core management group, each working group and platforms

**Platforms**

**Bioresource**

W Greenhalf (*Co-Lead*), M G Semple (*Co-Lead*), M Ashworth, H E Hardwick, L Lavelle-Langham, W Reynolds, M Sereno, R M Saunders, A Singapuri, V Shaw, A Shikotra, B Venson, L V Wain

**Data Hub**

A B Docherty (*Co-Lead*), E M Harrison (*Co-Lead*), A Sheikh (*Co-Lead*), J K Baillie, C E Brightling, L Daines, R Free, R A Evans, S Kerr, O C Leavy, N I Lone, D Lozano-Rojas, H J C McAuley, K Ntotsis, R Pius, J Quint, M Richardson, , M Sereno, M Thorpe, L V Wain

**Imaging Alliance**

M Halling-Brown (*Co-Lead*), F Gleeson (*Co-Lead*), J Jacob (*Co-Lead*), S Neubauer (*Co-Lead*) B Raman (*Co-Lead*) S Siddiqui (*Co-Lead*) J M Wild (*Co-Lead*), S Aslani, G Baxter, M Beggs, C Bloomfield, M P Cassar, A Chiribiri, E Cox, D J Cuthbertson, M Halling-Brown, V M Ferreira, L Finnigan, S Francis, P Jezzard, G J Kemp, H Lamlum, E Lukaschuk, C Manisty, G P McCann , C McCracken, K McGlynn , R Menke , C A Miller , A J Moss, T E Nichols, C Nikolaidou , C O’Brien , G Ogbole, B Rangelov, D P O’Regan , A Pakzad, S Piechnik , S Plein, I Propescu, A A Samat, L Saunders, Z B Sanders, R Steeds, T Treibel, E M Tunnicliffe, M Webster, J Willoughby, J Weir McCall, C Xie, M Xu

**Omics**

L V Wain (*Co-Lead)*, J K Baillie (*Co-Lead*), H Baxendale, C E Brightling, M Brown, J D Chalmers, R A Evans, B Gooptu, W Greenhalf, H E Hardwick, R G Jenkins, D Jones, I Koychev, C Langenberg, A Lawrie, P L Molyneaux, A Shikotra, J Pearl, M Ralser, N Sattar, R M Saunders, J T Scott, T Shaw, D Thomas, D Wilkinson

**Working Groups**

**Airways**

L G Heaney (*Co-Lead*), A De Soyza (*Co-Lead*), D Adeloye, C E Brightling, J S Brown, J Busby, J D Chalmers, C Echevarria, L Daines, O Elneima, RA Evans, J Hurst, P Novotny, C Nicolaou, P Pfeffer, K Poinasamy, J Quint, I Rudan, E Sapey, M Shankar-Hari, A Sheikh, S Siddiqui, S Walker, B Zheng

**Brain**

J R Geddes (*Lead*), M Hotopf *(Co-Lead),* K Abel, R Ahmed, L Allan, C Armour, D Baguley, D Baldwin, C Ballard, K Bhui, G Breen, K Breeze, M Broome, T Brugha, E Bullmore, D Burn, F Callard, J Cavanagh, T Chalder, D Clark, A David, B Deakin, H Dobson, B Elliott, J Evans, RA Evans, R Francis, E Guthrie, P Harrison, M Henderson,  A Hosseini, N Huneke, M Husain, T Jackson, I Jones, T Kabir, P Kitterick, A Korszun, I Koychev, J Kwan, A Lingford-Hughes, P Mansoori, H McAllister-Williams, K McIvor, B Michael, L Milligan, R Morriss, E Mukaetova-Ladinska, K Munro, A Nevado-Holgado, T Nicholson, C Nicolaou, S Paddick, C Pariante, J Pimm, K Saunders, M Sharpe, G Simons, J P Taylor, R Upthegrove, S Wessely

**Cardiac**

G P McCann (*Lead*), S Amoils, C Antoniades, A Banerjee, A Bularga, C Berry, P Chowienczyk, J P Greenwood, A D Hughes, K Khunti, C Lawson, N L Mills, A J Moss, S Neubauer, B Raman, A N Sattar, C L Sudlow, M Toshner,

**Immunology**

P J M Openshaw (*Lead*), D Altmann, J K Baillie, R Batterham, H Baxendale, N Bishop, C E Brightling, P C Calder, C M Efstathiou, R A Evans, J L Heeney, T Hussell, P Klenerman, F Liew, J M Lord, P Moss, S L Rowland-Jones, W Schwaeble, M G Semple, R S Thwaites, L Turtle, L V Wain, S Walmsley, D Wraith

**Intensive Care**

M J Rowland (*Lead*), A Rostron (*Co-Lead*), J K Baillie, B Connolly, A B Docherty, N I Lone, D F McAuley, D Parekh, A Rostron, J Simpson, C Summers

**Lung Fibrosis**

R G Jenkins (*Co-Lead*), J Porter (*Co-Lead*), R J Allen, R Aul, J K Baillie, S Barratt, P Beirne, J Blaikley, R C Chambers, N Chaudhuri, C Coleman, E Denneny, L Fabbri, P M George, M Gibbons, F Gleeson, B Gooptu, B Guillen Guio, I Hall, N A Hanley, L P Ho, E Hufton, J Jacob, I Jarrold, G Jenkins, S Johnson, M G Jones, S Jones, F Khan, P Mehta, J Mitchell, P L Molyneaux, J E Pearl, K Piper Hanley, K Poinasamy, J Quint, D Parekh, P Rivera-Ortega, L C Saunders, M G Semple, J Simpson, D Smith, M Spears, L G Spencer, S Stanel, I Stewart, A A R Thompson, D Thickett, R Thwaites, L V Wain, S Walker, S Walsh, J M Wild, D G Wootton, L Wright

**Metabolic**

S Heller (*Co-Lead*), M J Davies (*Co-Lead*), H Atkins, S Bain, J Dennis, K Ismail, D Johnston, P Kar, K Khunti, C Langenberg, P McArdle, A McGovern, T Peto, J Petrie, E Robertson, N Sattar, K Shah, J Valabhji, B Young

**Pulmonary and Systematic Vasculature**

L S Howard (*Co-Lead*), Mark Toshner (*Co-Lead*), C Berry, P Chowienczyk, A Lawrie, O C Leavy, J Mitchell, J Newman, L Price, J Quint, A Reddy, J Rossdale, N Sattar, C Sudlow, A A R Thompson, J M Wild, M Wilkins

**Rehabilitation, Sarcopenia and Fatigue**

S J Singh (*Co-Lead*), W D-C Man (*Co-Lead*), J M Lord (*Co-Lead*), N J Greening (*Co-Lead*), T Chalder (*Co-Lead*), J T Scott (*Co-Lead*), N Armstrong, E Baldry, M Baldwin, N Basu, M Beadsworth, L Bishop, C E Bolton, A Briggs, M Buch, G Carson, J Cavanagh, H Chinoy, C Dawson, E Daynes, S Defres, R A Evans, L Gardiner, P Greenhaff, S Greenwood, M Harvie, L HOuchen-Wolloff, M Husain, S MacDonald, A McArdle, H J C McAuley, A McMahon, M McNarry, G Mills, C Nolan, K O’Donnell, D Parekh, Pimm, J Sargent, L Sigfrid, M Steiner, D Stensel, A L Tan, I Vogiatzis, J Whitney, D Wilkinson, D Wilson, M Witham, D G Wootton, T Yates

**Renal**

D Thomas (*Lead*), N Brunskill (*Co-Lead*), S Francis (*Co-Lead*), S Greenwood (*Co-Lead*), C Laing (*Co-Lead*), K Bramham, P Chowdhury, A Frankel, L Lightstone, S McAdoo, K McCafferty, M Ostermann, N Selby, C Sharpe, M Willicombe

**Patient Public Engagement Group**

L Houchen-Wolloff (*Lead*), J Bunker, R Gill, C Hastie, R Nathu, N Rogers, N Smith

**Local Clinical Centre PHOSP-COVID trial staff**

(listed in alphabetical order)

**Airedale NHS Foundation Trust**

A Shaw (PI), L Armstrong, B Hairsine, H Henson, C Kurasz, L Shenton

**Aneurin Bevan University Health Board**

S Fairbairn (PI), A Dell, N Hawkings, J Haworth, M Hoare, A Lucey, V Lewis, G Mallison, H Nassa, C Pennington, A Price, C Price, A Storrie, G Willis, S Young

**Barts Health NHS Trust &** **Queen Mary University of London**

P Pfeffer (PI), K Chong-James, C David, W Y James, C Manisty, A Martineau, O Zongo

**Barnsley Hospital NHS Foundation Trust**

A Sanderson (PI)

**Belfast Health and Social Care Trust & Queen's University Belfast**

L G Heaney (PI), C Armour, V Brown, T Craig, S Drain, B King, N Magee, D McAulay, E Major, L McGarvey, J McGinness, R Stone

**Betsi Cadwaladr University Health Board**

A Haggar (PI), A Bolger, F Davies, J Lewis, A Lloyd, R Manley, E McIvor, D Menzies, K Roberts, W Saxon, D Southern, C Subbe, V Whitehead

**Borders General Hospital, NHS Borders**

H El-Taweel (PI), J Dawson, L Robinson

**Bradford Teaching Hospitals NHS Foundation Trust**

D Saralaya (PI), L Brear, K Regan, K Storton

**Cambridge University Hospitals NHS Foundation Trust, NIHR Cambridge Clinical Research Facility & University of Cambridge**

J Fuld (PI), A Bermperi, I Cruz, K Dempsey, A Elmer, H Jones, S Jose, S Marciniak, M Parkes, C Ribeiro, J Taylor, M Toshner, L Watson, J Weir McCall, J Worsley

**Cardiff and Vale University Health Board**

R Sabit (PI), L Broad, A Buttress, T Evans, M Haynes, L Jones, L Knibbs, A McQueen, C Oliver, K Paradowski, J Williams

**Chesterfield Royal Hospital NHS Trust**

E Harris (PI), C Sampson

**Cwm Taf Morgannwg University Health Board**

C Lynch (PI), E Davies, C Evenden , A Hancock, K Hancock, M Rees , L Roche, N Stroud, T Thomas-Woods

**East Cheshire NHS Trust**

M Babores (PI), J Bradley-Potts, M Holland, N Keenan, S Shashaa, H Wassall

**East Kent Hospitals University NHS Foundation Trust**

E Beranova (PI), H Weston (PI), T Cosier, L Austin, J Deery, T Hazelton, C Price, H Ramos, R Solly, S Turney

**Gateshead NHS Trust**

L Pearce (PI), W McCormick, S Pugmire, W Stoker, A Wilson

**Guy’s and St Thomas’ NHS Foundation Trust**

N Hart (PI), LA Aguilar Jimenez, G Arbane, S Betts, K Bisnauthsing, A Dewar, P Chowdhury, A Chiribiri, A Dewar, G Kaltsakas, H Kerslake, MM Magtoto, P Marino, LM Martinez, C O'Brien, M Ostermann, J Rossdale, TS Solano, E Wynn

**Hampshire Hospitals NHS Foundation Trust**

N Williams (PI), W Storrar (PI), M Alvarez Corral, A Arias, E Bevan, D Griffin, J Martin, J Owen,

S Payne, A Prabhu, A Reed, C Wrey Brown

**Harrogate and District NHD Foundation Trust**

C Lawson (PI), T Burdett, J Featherstone, A Layton, C Mills, L Stephenson,

**Hull University Teaching Hospitals NHS Trust & University of Hull**

N Easom (PI), P Atkin, K Brindle, M G Crooks, K Drury, R Flockton, L Holdsworth, A Richards, D L Sykes, S Thackray-Nocera, C Wright

**Hywel Dda University Health Board**

K E Lewis (PI), A Mohamed (PI), G Ross (PI), S Coetzee, K Davies, R Hughes, R Loosley, L O’Brien, Z Omar, H McGuinness, E Perkins, J Phipps, A Taylor, H Tench, R Wolf-Roberts

**Imperial College Healthcare NHS Trust & Imperial College London**

L S Howard (PI), O Kon (PI), D C Thomas (PI), S Anifowose, L Burden, E Calvelo, B Card, C Carr, E R Chilvers, D Copeland, P Cullinan, P Daly, C M Efstathiou, L Evison, T Fayzan, H Gordon, S Haq, R G Jenkins, C King, F Liew, K March, M Mariveles, L McLeavey, N Mohamed, S Moriera, U Munawar, J Nunag, U Nwanguma, L Orriss- Dib, D P O'Regan, A Ross, M Roy, E Russell, K Samuel, J Schronce, N Simpson, L Tarusan, C Wood, N Yasmin

**Kettering General Hospital NHS Trust**

R Reddy (PI), A-M, Guerdette, M Hewitt, K Warwick, S White

**King’s College Hospital NHS Foundation Trust & Kings College London**

A M Shah (PI), C J Jolley (PI), O Adeyemi, R Adrego, H Assefa-Kebede, J Breeze, M Brown, S Byrne, T Chalder, A Chiribiri, P Dulawan, N Hart, A Hayday, A Hoare, A Knighton, M Malim, C O'Brien, S Patale, I Peralta, N Powell, A Ramos, K Shevket, F Speranza, A Te

**Leeds Teaching Hospitals & University of Leeds**

P Beirne (PI), A Ashworth, J Clarke, C Coupland, M Dalton, E Wade, C Favager, J Greenwood, J Glossop, L Hall, T Hardy, A Humphries, J Murira, D Peckham, S Plein, J Rangeley, G Saalmink, A L Tan, B Whittam, N Window, J Woods,

**Lewisham & Greenwich NHS Trust**

G Coakley (PI)

**Liverpool University Hospitals NHS Foundation Trust & University of Liverpool**

D G Wootton (PI), L Turtle (PI), L Allerton, AM All, M Beadsworth, A Berridge, J Brown, S Cooper, A Cross, D J Cuthbertson, S Defres, S L Dobson, J Earley, N French, W Greenhalf, H E Hardwick, K Hainey, J Hawkes, V Highett, S Kaprowska, G J Kemp, AL Key, S Koprowska, L Lavelle-Langham, N Lewis-Burke, G Madzamba, F Malein, S Marsh, C Mears, L Melling, M J Noonan, L Poll, J Pratt, E Richardson, A Rowe, M G Semple, V Shaw, K A Tripp, B Vinson, L O Wajero, S A Williams-Howard, J Wyles

**London North West University Healthcare NHS Trust**

S N Diwanji (PI), P Papineni (PI), S Gurram, S Quaid, G F Tiongson, E Watson

**Manchester University NHS Foundation Trust & University of Manchester**

B Al-Sheklly (PI), A Horsley (PI), C Avram, P Barran, J Blaikely, M Buch, N Choudhury, D Faluyi, T Felton, T Gorsuch, N A Hanley, T Hussell, Z Kausar, C A Miller, N Odell, R Osbourne, K Piper Hanley, K Radhakrishnan, S Stockdale, D Trivedi

**Newcastle upon Tyne Hospitals NHS Foundation Trust & University of Newcastle**

A De Soyza (PI), C Echevarria (PI), A Ayoub, J Brown, G Burns, G Davies, H Fisher, C Francis, A Greenhalgh, P Hogarth, J Hughes, K Jiwa, G Jones, G MacGowan, D Price, A Sayer, J Simpson, H Tedd, S Thomas, S West, M Witham, S Wright, A Young

**NHS Dumfries and Galloway**

M J McMahon (PI), P Neill

**NHS Greater Glasgow and Clyde Health Board & University of Glasgow**

D Anderson (PI), H Bayes (PI), C Berry (PI), D Grieve (PI), I B McInnes (PI), N Basu, A Brown, A Dougherty, K Fallon, L Gilmour, K Mangion, A Morrow, K Scott, R Sykes, R Touyz

**NHS Highland**

E K Sage (PI), F Barrett, A Donaldson

**NHS Lanarkshire**

M Patel (PI), D Bell, A Brown, M Brown, R Hamil, K Leitch, L Macliver, J Quigley, A Smith, B Welsh

**NHS Lothian & University of Edinburgh**

G Choudhury (PI), J K Baillie, S Clohisey, A Deans, A B Docherty, J Furniss, E M Harrison, S Kelly, N I Lone, D E Newby, A Sheikh

**NHS Tayside & University of Dundee**

J D Chalmers (PI), D Connell, A Elliott, C Deas, J George, S Mohammed, J Rowland, A R Solstice, D Sutherland, C J Tee

**North Bristol NHS Trust & University of Bristol**

N Maskell (PI), D Arnold, S Barrett, H Adamali, A Dipper, S Dunn, A Morley, L Morrison, L Stadon, S Waterson, H Welch

**North Middlesex Hospital NHS Trust**

B Jayaraman (PI), T Light

**Nottingham University Hospitals NHS Trust & University of Nottingham**

C E Bolton (PI), P Almeida, J Bonnington, M Chrystal, E Cox, C Dupont, S Francis, P Greenhaff, A Gupta, L Howard, W Jang, S Linford, L Matthews, R Needham, A Nikolaidis, S Prosper, K Shaw, A K Thomas

**Oxford University Hospitals NHS Foundation Trust & University of Oxford**

L P Ho (PI), N M Rahman (PI), M Ainsworth, A Alamoudi, M Beggs, A Bates, A Bloss, A Burns, P Carter, M Cassar, K M Channon, J Chen, F Conneh, T Dong, R I Evans, E Fraser, X Fu, J R Geddes, F Gleeson, P Harrison, M Havinden-Williams, P Jezzard, N Kanellakis, I Koychev, P Kurupati, X Li, E Lukaschuk, K McGlynn, H McShane, C Megson, K Motohashi, S Neubauer, D Nicoll, G Ogg, E Pacpaco, M Pavlides, Y Peng, N Petousi, J Propescu, N Rahman, B Raman, M J Rowland, K Saunders, M Sharpe, N Talbot, E Tunnicliffe

**Royal Brompton and Harefield Clinical Group, Guy’s and St Thomas’ NHS Foundation Trust.**

W D-C Man (PI), B Patel (PI), R E Barker, D Cristiano, N Dormand, M Gummadi, S Kon, K Liyanage, C M Nolan, S Patel, O Polgar, P Shah, S J Singh, J A Walsh

**Royal Free London NHS Foundation Trust**

J Hurst (PI), H Jarvis (PI), S Mandal (PI), S Ahmad, S Brill, L Lim, D Matila, O Olaosebikan, C Singh

**Royal Papworth Hospital NHS Foundation Trust**

M Toshner (PI), H Baxendale, L Garner, C Johnson, J Mackie, A Michael, J Pack, K Paques, H Parfrey, J Parmar

**Salford Royal NHS Foundation Trust**

N Diar Bakerly (PI), P Dark, D Evans, E Hardy, A Harvey, D Holgate, S Knight, N Mairs, N Majeed, L McMorrow, J Oxton, J Pendlebury, C Summersgill, R Ugwuoke, S Whittaker

**Salisbury NHS Foundation Trust**

W Matimba-Mupaya (PI), S Strong-Sheldrake

**Sheffield Teaching NHS Foundation Trust & University of Sheffield**

S L Rowland-Jones (PI), A A R Thompson (Co PI), J Bagshaw, M Begum, K Birchall, R Butcher, H Carborn, F Chan, K Chapman, Y Cheng, L Chetham, C Clark, Z Coburn, J Cole, M Dixon, A Fairman, J Finnigan, L Finnigan, H Foot, D Foote, A Ford, R Gregory, K Harrington, L Haslam, L Hesselden, J Hockridge, A Holbourn, B Holroyd-Hind, L Holt, A Howell, E Hurditch, F Ilyas, C Jarman, A Lawrie, E Lee, J-H Lee, R Lenagh, A Lye, I Macharia, M Marshall, A Mbuyisa, J McNeill, S Megson, J Meiring, L Milner, S Misra, H Newell, T Newman, C Norman, L Nwafor, D Pattenadk, M Plowright, J Porter, P Ravencroft, C Roddis, J Rodger, P Saunders, J Sidebottom, J Smith, L Smith, N Steele, G Stephens, R Stimpson, B Thamu, N Tinker, K Turner, H Turton, P Wade, S Walker, J Watson, J M Wild, I Wilson, A Zawia

**St George’s University Hospitals NHS Foundation Trust**

R Aul (PI), M Ali, A Dunleavy (PI), D Forton, N Msimanga, M Mencias, T Samakomva, S Siddique, J Teixeira, V Tavoukjian

**Sherwood Forest Hospitals NHS Foundation Trust**

J Hutchinson (PI), L Allsop, K Bennett, P Buckley, M Flynn, M Gill, C Goodwin, M Greatorex, H Gregory, C Heeley, L Holloway, M Holmes, J Kirk, W Lovegrove, TA Sewell, S Shelton, D Sissons, K Slack, S Smith, D Sowter, S Turner, V Whitworth, I Wynter

**Shropshire Community Health NHS Trust**

L Warburton (PI), S Painter, J Tomlinson

**Somerset NHS Foundation Trust**

C Vickers (PI), T Wainwright, D Redwood, J Tilley, S Palmer

**Swansea Bay University Health Board**

G A Davies (PI), L Connor, A Cook, T Rees, F Thaivalappil, C Thomas

**Tameside and Glossop Integrated Care NHS Foundation**

A Butt (PI), M Coulding, H Jones, S Kilroy, J McCormick, J McIntosh, H Savill, V Turner, J Vere

**The Great Western Hospital Foundation Trust**

E Fraile (PI), J Ugoji

**The Hillingdon Hospitals NHS Foundation Trust**

S S Kon (PI), H Lota, G Landers, M Nasseri, S Portukhay

**The Rotherham NHS Foundation Trust**

A Hormis (PI), A Daniels, J Ingham, L Zeidan

**United Lincolnshire Hospitals NHS Trust**

M Chablani (PI), L Osborne

**University College London Hospital & University College London**

M Marks (PI), J S Brown (PI), N Ahwireng, B Bang, D Basire, R C Chambers, A Checkley, R Evans, M Heightman, T Hillman, J Hurst, J Jacob, S Janes, R Jastrub, M Lipman, S Logan, D Lomas, M Merida Morillas, A Pakzad, H Plant, J C Porter, K Roy, E Wall, B Williams, M Xu

**University Hospital Birmingham NHS Foundation Trust & University of Birmingham**

D Parekh (PI), N Ahmad Haider, C Atkin, R Baggott, M Bates, A Botkai, A Casey, B Cooper, J Dasgin, K Draxlbauer, N Gautam, J Hazeldine, T Hiwot, S Holden, K Isaacs, T Jackson, S Johnson, V Kamwa, D Lewis,

J M Lord, S Madathil, C McGhee, K Mcgee, A Neal, A Newton Cox, J Nyaboko, D Parekh, Z Peterkin, H Qureshi, B Rangelov, L Ratcliffe, E Sapey, J Short, T Soulsby, R Steeds, J Stockley, Z Suleiman, T Thompson, M Ventura, S Walder, C Welch, D Wilson, S Yasmin, K P Yip

**University Hospitals of Derby and Burton**

P Beckett (PI) C Dickens, U Nanda

**University Hospitals of Leicester NHS Trust & University of Leicester**

C E Brightling (CI), R A Evans (PI), M Aljaroof, N Armstrong, H Arnold, H Aung, M Bakali, M Bakau, M Baldwin, M Bingham, M Bourne, C Bourne, N Brunskill, P Cairns, L Carr, A Charalambou, C Christie, M J Davies, S Diver, S Edwards, C Edwardson, O Elneima, H Evans, J Finch, S Glover, N Goodman, B Gootpu, N J Greening, K Hadley, P Haldar, B Hargadon, V C Harris, L Houchen-Wolloff, W Ibrahim, L Ingram, K Khunti, A Lea, D Lee, D Lozano-Rojas, G P McCann, H J C McAuley, P McCourt, T Mcnally, G Mills, A Moss, W Monteiro, K Ntotsis, M Pareek, S Parker, A Rowland, A Prickett, I N Qureshi, R Russell, N Samani, M Sereno, M Sharma, A Shikotra, S Siddiqui, A Singapuri, S J Singh, J Skeemer, M Soares, E Stringer, T Thornton, M Tobin, E Turner, L V Wain, T J C Ward, F Woodhead, J Wormleighton, T Yates, A Yousuf,

**University Hospital Southampton NHS Foundation Trust & University of Southampton**

M G Jones (PI), C Childs, R Djukanovic, S Fletcher, M Harvey, E Marouzet, B Marshall, R Samuel, T Sass, T Wallis, H Wheeler

**Whittington Health NHS**

R Dharmagunawardena (PI), E Bright, P Crisp, M Stern

**Wirral University Teaching Hospital**

A Wight (PI), L Bailey, A Reddington

**Wrightington Wigan and Leigh NHS trust**

A Ashish (PI), J Cooper, E Robinson

**Yeovil District Hospital NHS Foundation Trust**

A Broadley (PI)

**York & Scarborough NHS Foundation Trust**

K Howard (PI), L Barman, C Brookes, K Elliott. L Griffiths, Z Guy, D Ionita, H Redfearn, C Sarginson

A Turnbull

**Health and Care Research Wales**

Y Ellis

**London School of Hygiene & Tropical Medicine (LSHTM)**

M Marks, A Briggs

**NIHR Office for Clinical Research Infrastructure**

K Holmes

**Patient Public Involvement Leads**

Asthma UK and British Lung Foundation Partnership - K Poinasamy, S Walker

**Royal Surrey NHS Foundation Trust**

M Halling-Brown

**South London and Maudsley NHS Foundation Trust & Kings College London**

G Breen, M Hotopf

**Swansea University & Swansea Welsh Network**

K Lewis, N Williams

# Table S1- Outcome measures

| **Module** | **Tier 2 outcome measures reported in the**  **current analysis** | **Other Tier 2 outcome measures not analysed** |
| --- | --- | --- |
| **Symptoms** | Patient symptom questionnaire (PHOSP-COVID study specific questionnaire)  Dyspnoea12 Questionnaire  The Functional Assessment of Chronic Illness Therapy (FACIT) | MRC dyspnoea scale grade Nottingham activities of daily living Questionnaire  Brief Pain Inventory Questionnaire (BPI) |
| **Health-related Quality of**  **life and Disability** | Euroqol EQ5D-5L  Washington Short Set of Functioning |  |
| **Respiratory** | Pulmonary Function Tests Including: Spirometry  (FEV_1_, FVC) | Transfer Factor (TLCO, KCO) |
| **Cardiac** |  | ECG Image Collection  Blood tests: Troponin I /Troponin T & Lipid Profile  Blood tests: BNP / NT-Pro-BNP |
| **Renal** | Blood tests: eGFR | Urine tests: Albumin: Creatinine Ratio, Protein: Creatinine Ratio, Bedside urinalysis |
| **Pre-diabetes/diabetes** | Blood tests: HbA1C |  |
| **Hematological** |  | Blood tests: Full Blood Count, INR  Blood tests: D Dimer, Ferritin |
| **Systemic inflammation** | Blood test: CRP | Blood tests: Fibrinogen |
| **Other organ function** |  | Blood tests: Liver function tests, 25-  Hydroxyvitamin D, Bone Profile |
| **Physical performance** | Incremental Shuttle Walk Test (ISWT) to assess exercise capacity | Daily physical activity by wearable technology (Geneactive)  Handgrip Strength  General Practice Physical Activity Questionnaire (GPPAQ)  Short Physical Performance Battery (SPPB) |
| **Frailty** | Rockwood Clinical Frailty Scale (CFS) | Fried’s frailty definition  SARC-F Questionnaire |
| **Body composition** | Body Mass Index (BMI) calculation from Height and Weight Measurement | Body composition estimation via: Bio-Electrical Impedance Analysis (BIA) or Duel Energy X-ray Analysis (DXA)  Waist Circumference Measurement |
| **Mental Health** | Generalised Anxiety Disorder Questionnaire (GAD-7)  Patient Health Questionnaire (PHQ-9) |  |
| **Cognition** | Montreal Cognitive Assessment (MoCA) |  |

FEV_1_ = Forced Expiratory Volume in 1 second, FVC = Forced Vital Capacity, TLCO = Transfer Capacity of the Lung for Carbon Monoxide, KCO = carbon monoxide transfer coefficient, BNP = Brain Natriuretic Peptide or NT-BNP N-Terminal Brain Natriuretic Peptide, ECG = electrocardiogram, HbA1C = glycosylated haemoglobin, INR = international normalized ratio, eGFR = estimated Glomerular Filtration Rate, CRP = C - reactive protein

# Table S2. Methods and thresholds for processing of variables and outcome measures

|  | **Method** |
| --- | --- |
| **Table 1** |  |
| Indices of Multiple Deprivation | Obtained using postcode.[1] |
| Comorbidities | A pre-existing comorbidity was considered absent if not indicated by a ‘yes’ on the case report form. |
| Admission duration | Calculated using the hospital discharge date and the earliest admission date to the same or different hospital for the participant’s COVID-19 episode. |
| **Table 2 and 3** |  |
| Symptoms at five months and one year | The total number of current symptoms reported were from the following list which were answered as binary Yes/No questions: Aching in your muscles (pain), Physical slowing down, Slowing down in your thinking, Joint pain or swelling, Limb weakness, Difficulty with concentration, Short term memory loss, Headache, Tingling feeling/pins and needles, Confusion/fuzzy head, Dizziness or light headedness, Chest tightness, Problems with balance, Altered personality/ behaviour, Chest pain, Palpitations, Leg/ankle swelling, Difficulty with communication, Skin rash, Diarrhoea, Problems seeing, Pain on breathing, Weight loss, Tremor/shakiness, Constipation, Erectile Dysfunction, Loss of sense of smell, Can’t fully move or control movement, Abdominal pain, Stomach pain, Loss of control of passing urine, Loss of appetite, Loss of taste, Nausea/vomiting, Bleeding, Can’t move and/or feel one side of your body or face, Loss of control of opening bowels, Lumpy lesions on toes, Fainting / blackouts, Seizures  Symptom severity was rated using a 0-10 visual analogue scale for Breathlessness, Cough, Fatigue, Sleep quality and Pain before COVID-19 illness and worst in last 24 hours. |
| Generalised Anxiety Disorder Questionnaire (GAD-7) (Anxiety) | The Generalised Anxiety Disorder (GAD-7) questionnaire is a patient reported outcome measure consists of 7 questions with total scores ranging from 0 to 21. We used a GAD7 threshold score of > 8 to suggest at least mild-moderate anxiety.[2] |
| Dyspnoea-12 | The Dyspnoea-12 questionnaire is a patient reported outcome measure consisting of 12 questions assessing breathlessness severity incorporating both “physical” and “affective” aspects.[3] Scores range from 0 to 36 with higher scores correspond to greater severity of breathlessness. |
| FACIT fatigue subscale score (FACIT) | The Functional Assessment of Chronic Illness Therapy – Fatigue (FACIT-Fatigue) scale is a patient reported outcome measure consisting of 13 questions to assess self-reported fatigue and its impact on  daily activities and function.[4] Total scores range from 0-52, with lower scores corresponding to an increased burden of fatigue.[5] |
| Brief Pain Inventory (BPI) severity and interference | The Brief Pain Inventory (BPI) is a patient reported outcome questionnaire consisting of 15 questions across domains of pain severity and pain interference. We have reported the BPI Severity score as the mean score from the 4 severity questions each with a range 0 – 10 anchored at 0 = “No Pain” and 10 =  “Pain as bad as you can imagine”.[6, 7] |
| Short Physical Performance Battery (SPPB) | The Short Physical Performance Battery (SPPB) test is a researcher administer assessment of physical performance and frailty. It comprises 3 components; balance, gait speed and sit to stand tests. Tests were completed according to recommended standards and training was provided to site staff by the central study team via a recorded demonstration video. SPPB total scores range from 0-12. We have reported a  total SPPB score of ≤10 suggestive of underlying frailty.[8-10] |
| Incremental Shuttle Walk Test (ISWT) | The Incremental Shuttle Walk Test (ISWT) is a researcher administered assessment of maximal physical performance and was performed according to standardised instructions with two attempts performed by participants on the same day with a 20 minutes rest between them.[11] Training was provided to site staff by the central study team via a recorded demonstration video. The best effort was reported in metres and the percent predicted value was calculated using the following reference formula accounting for gender, age and BMI.[12] (ISWT predicted = 1449·701 − (11·735 × age) + (241·897 × gender) − (5·686 × BMI), where male gender = 1 and female gender = 0) |
| Rockwood Clinical Frailty Scale (CFS) | The Rockwood Clinical Frailty Scale (CFS) is a researcher assessed scale of clinical frailty with scores ranging from 1-9 where lower scores correspond to increased frailty. We have reported CFS scores of <5  suggestive of frailty.[13] |
| Montreal Cognitive Assessment (MoCA) | The Montreal Cognitive Assessment (MoCA) is a researcher administered cognitive function questionnaire across 8 domains. Training was provided to site staff using standardised resources supplied online by MoCA TEST Inc.[14] The assessment was conducted in English with researchers applying their discretion to exclude participants whose command of English was insufficient to complete the test accurately. Total scores range from 0 to 30. We report total MoCA scores of <23 suggestive of at least  Mild Cognitive Impairment.[15] |
| Spirometry and Pulmonary Function Testing | Due to COVID-19 related restrictions on aerosol-generating procedures during the study period, access to  spirometry and lung function was limited. Spirometry and Pulmonary function testing was completed as per ERS/ATS recommendations.[16] Spirometry were converted to SI units if not reported as such by sites. |
| BNP / NT-pro BNP | Brain Natriuretic Peptide (BNP) or N-terminal pro B-type Natriuretic Peptide (NT-pro BNP) were collected by according to each site’s routine clinically available assay as a biomarker of heart failure. Three sites submitted BNP results with all of the remaining sites submitting NT-pro BNP results. The threshold values used for BNP was ≥ 100 ng/litre [17] and for NT-pro BNP ≥ 400ng/litre [18] as suggestive of heart failure. |
| Glycated haemoglobin (HbA1c) | Glycated haemoglobin (HbA1c) was collected as a biomarker of current glycaemic control. We have  reported HbA1c levels ≥ 6·5% as suggestive of a diagnosis of diabetes.[19] |
| C-Reactive Protein (CRP) | C-Reactive Protein (CRP) levels were collected as a biomarker of current systemic inflammation. Values reported as below the lower or upper limit reportable range for the assay used at the site have been  included at the stated less than or more than cut off value for calculation of mean (SD) results. We have reported CRP levels > 5mg/L as suggestive of systemic inflammation. |
| EQ5D-5L VAS | The EQ5D Visual Analogue Scale is a patient reported outcome questionnaire recording the patient’s self-  rated health and was completed for “before your COVID-19 illness” and “your own health state today.” Scores are presented as mean and standard deviation.[20] |
| EQ5D-5L Utility Index | The EQ5D-5L is a five-dimension patient reported outcome questionnaire recording a patient’s self-rated health state for mobility, self-care, usual activities, pain/discomfort and anxiety/depression. These scores are then mapped to a United Kingdom specific Utility Index anchored at 1 for “perfect health” and 0 for  “dead” calculated from reported EQ5D-5L scores across the five dimensions.[21] |
| Washington Group Short Set of Functioning Severity Continuum | The Washington Group Short Set of Functioning (WG-SS) is a patient reported outcome questionnaire using six questions to assess disability and function. Participant responses were transformed to the  “Severity Continuum” by assigning scores of zero to responses “no difficulty”, one to responses “some difficulty”, six to responses “a lot of difficulty” and 36 to responses “cannot do at all”. [22] |

# Table S3 – PHOSP participants’ characteristics by tier

|  |  | **Total** | **without DM** | **with DM** | **p-value** |
| --- | --- | --- | --- | --- | --- |
|  |  | **N=7,768** | **N=5,223** | **N=2,545** |  |
| Age at admission (years) |  | 59.3 (13.4) | 59.9 (13.8) | 58.0 (12.6) | <0.0001 |
| Sex at birth | Female | 3,154 (40.6%) | 2,166 (41.5%) | 988 (38.8%) | 0.026 |
|  | Male | 4,614 (59.4%) | 3,057 (58.5%) | 1,557 (61.2%) |  |
| Ethnicity | White | 6,175 (81.9%) | 4,282 (85.5%) | 1,893 (74.8%) | <0.0001 |
|  | South Asian | 423 (5.6%) | 225 (4.5%) | 198 (7.8%) |  |
|  | Black | 362 (4.8%) | 182 (3.6%) | 180 (7.1%) |  |
|  | Mixed/other | 580 (7.7%) | 321 (6.4%) | 259 (10.2%) |  |
| Diabetes (1 or 2) |  | 1,732 (22.3%) | 1,194 (22.9%) | 538 (21.1%) | 0.087 |
| No. comorbidities | 0 | 3,468 (44.6%) | 2,274 (43.5%) | 1,194 (46.9%) | 0.023 |
|  | 1 | 1,836 (23.6%) | 1,250 (23.9%) | 586 (23.0%) |  |
|  | 2 | 1,269 (16.3%) | 862 (16.5%) | 407 (16.0%) |  |
|  | >2 | 1,195 (15.4%) | 837 (16.0%) | 358 (14.1%) |  |
| IHD |  | 610 (7.9%) | 438 (8.4%) | 172 (6.8%) | 0.011 |
| MI |  | 368 (4.7%) | 260 (5.0%) | 108 (4.2%) | 0.15 |
| HF |  | 196 (2.5%) | 155 (3.0%) | 41 (1.6%) | <0.0001 |
| AF/flutter |  | 468 (6.0%) | 350 (6.7%) | 118 (4.6%) | <0.0001 |
| Hypertension |  | 2,886 (37.2%) | 2,008 (38.4%) | 878 (34.5%) | <0.0001 |
| Congenital heart disease |  | 39 (0.5%) | 26 (0.5%) | 13 (0.5%) | 0.94 |
| Valve disease |  | 140 (1.8%) | 100 (1.9%) | 40 (1.6%) | 0.29 |
| Pacemaker/ICD |  | 108 (1.4%) | 87 (1.7%) | 21 (0.8%) | <0.0001 |
| Peripheral vascular disease |  | 104 (1.3%) | 68 (1.3%) | 36 (1.4%) | 0.69 |
| Hypercholesterolemia |  | 1,270 (16.3%) | 770 (14.7%) | 500 (19.6%) | <0.0001 |
| Other cardiac condition |  | 206 (2.7%) | 150 (2.9%) | 56 (2.2%) | 0.084 |
| CVA/TIA |  | 305 (3.9%) | 198 (3.8%) | 107 (4.2%) | 0.38 |
| Chronic Kidney Disease |  | 429 (5.5%) | 322 (6.2%) | 107 (4.2%) | <0.0001 |
| Time from 1st symptom to admission (days) |  | 7.0 (5.0-11.0) | 7.0 (4.0-10.0) | 8.0 (6.0-11.0) | <0.0001 |
| Admission duration (days) |  | 8.0 (4.0-15.0) | 8.0 (4.0-14.0) | 8.0 (4.0-16.0) | 0.63 |
| PCR positive test |  | 6,714 (94.0%) | 4,550 (94.5%) | 2,164 (93.0%) | 0.021 |
| WHO Clinical Progression Scale | Class 3/4 (no continuous O2) | 1,246 (16.7%) | 854 (17.1%) | 392 (15.9%) | <0.0001 |
|  | Class 5 (continuous O2) | 3,374 (45.2%) | 2,319 (46.4%) | 1,055 (42.7%) |  |
|  | Class 7 (cpap or bpap or high flow O2) | 1,858 (24.9%) | 1,274 (25.5%) | 584 (23.7%) |  |
|  | Class 7-9 (imv or ecmo) | 993 (13.3%) | 556 (11.1%) | 437 (17.7%) |  |
| Proning required |  | 1,356 (19.5%) | 887 (19.0%) | 469 (20.5%) | 0.14 |
| Renal replacement therapy |  | 263 (3.6%) | 159 (3.2%) | 104 (4.3%) | 0.021 |
| Pulmonary embolism |  | 606 (8.1%) | 364 (7.2%) | 242 (9.9%) | <0.0001 |
| Renal failure requiring haemodialysis |  | 194 (2.6%) | 112 (2.2%) | 82 (3.4%) | <0.0001 |
| Antibiotics |  | 6,029 (79.7%) | 4,078 (80.3%) | 1,951 (78.6%) | 0.087 |
| Systemic steroids |  | 4,534 (61.4%) | 3,147 (63.4%) | 1,387 (57.3%) | <0.0001 |
| Anticoagulation |  | 3,540 (48.4%) | 2,436 (49.8%) | 1,104 (45.5%) | <0.0001 |
| Lowest eGFR (ml/min/1.73m2) |  | 78.0 (60.0-91.0) | 76.0 (58.0-91.0) | 80.5 (61.0-91.0) | <0.0001 |
| Alanine Transaminase (U/L) |  | 54.0 (31.0-100.0) | 51.0 (29.0-94.0) | 60.0 (34.0-109.0) | <0.0001 |

Data are presented as mean (SD) or median (IQR) for continuous measures, and n (%) for categorical measures.

# Table S4- Linear Regression Models for Patient Reported Outcomes at 12-Months; Hierarchical Adjustment of 5-Month Measurements

|  | 12-month | | | | | | |  |
| --- | --- | --- | --- | --- | --- | --- | --- | --- |
| Outcome  (12-month measurement) | Model 1 | p-value | Model 2 | p-value | Model 3 | p-value | Model 4 | p-value |
| Fatigue FACIT | -1.42(-2.51, -0.33) | 0.011 | -1.46(-2.58, -0.33) | 0.018 | -1.15(-2.55, 0.22) | 0.12 | -1.19(-2.86, 0.48) | 0.16 |
| Fatigue VAS-now | 0.14(-0.25, 0.53) | 0.48 | 0.18( -0.21, 0.58) | 0.35 | -0.08(-0.62, 0.43) | 0.75 | 0.04(-0.55, 0.65) | 0.87 |
| EQ-5D now | -3.58(-6.10, -1.07) | 0.0017 | -3.28(-5.79, -0.76) | 0.013 | -2.2(-5.13, 0.70) | 0.13 | -3.45(-7.08, 0.17) | 0.066 |
| ISWT distance (m) | -20.15(-44.02, 3.72) | 0.095 | -15.95(-40.65, 8.74) | 0.21 | -16.83(-46.51, 12.83) | 0.26 | 7.00(-26.55, 40.55) | 0.68 |

Abbreviations: Fatigue (FACIT): fatigue (Functional Assessment of Chronic Illness Therapy; Fatigue VAS: Fatigue Visual Analogue Scale; EQ-5D-5L: EuroQol-5 Dimensions-5 Levels; ISWT distance: incremental shuttle walk test distance. *Comparing patients with diabetes vs. without diabetes

*Model 1: adjusted for age, sex; Model2: adjusted for age, sex, index of multiple deprivation, Ethnicity, and education; Model3: adjusted for Model2 + BMI; Model4: Model3+number of long-term conditions*

# Table S5- Logistic Regression for Patient Reported Outcomes at 12-Months; Hierarchical Adjustment of 5-Month Measurements

|  | OR (95%CI) | | | | | | | |
| --- | --- | --- | --- | --- | --- | --- | --- | --- |
| Outcome  (12-month measurement) | Model 1 | p-value | Model 2 | p-value | Model 3 | p-value | Model 4 | p-value |
| MoCA score<23 | 1.66(1.07, 2.58) | 0.023 | 1.39(0.89, 2.18) | 0.14 | 1.20(0.71, 2.04) | 0.48 | 1.20(0.71, 2.04) | 0.48 |
| Anxiety (GAD-7 score >8 | 0.75(0.34, 1.64) | 0.47 | 1.00(0.68, 1.49) | 0.96 | 0.92(0.61, 1.38) | 0.68 | 0.70(0.41, 1.22) | 0.21 |

Abbreviations: MoCA: The Montreal Cognitive Assessment; GAD-7: Generalised Anxiety Disorder 7-item scale. *Comparing patients with diabetes vs. without diabetes

*Model 1: adjusted for age, sex; Model2: adjusted for age, sex, index of multiple deprivation, Ethnicity, and education; Model3: adjusted for Model2 + BMI; Model4: Model3+number of long-term conditions*

# Table S6- Outcome change score from 5 to 12 months

|  | Total population | p-value** | Patients with DM | p-value | Patients without DM | p-value |
| --- | --- | --- | --- | --- | --- | --- |
| GAD scores | 0.0(-2.0, 2.0) | 0.35 | 0.0(-2.0, 2.0) | 0.52 | 0.0(-2.0, 2.0) | 0.41 |
| MOCA scores | 0.0(-1.0, 2.0) | 0.0031 | 0.0(-1.0, 2.0) | <0.0001 | 0.0(-1.0, 2.0) | <0.0001 |
| Fatigue FACIT | 0.0 (-2.0, 1.0) | <0.0001 | 0.0 (-2.0, 1.0) | 0.93 | 0.0 (-2.0, 1.0) | <0.0001 |
| Fatigue VAS | 0.0 (4.0 to 5.0) | <0.0001 | 0.0(-4.9 to 5.0) | 0.14 | 1.0 ( -3.0 to 5.0) | <0.0001 |
| ISWT distance (m) | 20.0(-40.0, 90.0) | 0.0041 | 20.0(-40.0, 80.0) | 0.0018 | 20.0 (-40.0, 100.0) | <0.0001 |
| EQ-5D | 0.0(-10.0, 10.0) | 0.25 | 0.0(-10.0, 10.0) | 0.35 | 0.0(-10.0, 10.0) | 0.072 |

*Change score= 12-month measurement – 5-month measurement; ** Wilcoxon signed rank test for paired data

# Table S7- Odds Ratio (with 95% confidence intervals) of diabetes in all four models, adjusted for admission to ITU at baseline.

|  | 5-month | | | | | | | | 12-Month | | | | | | | |
| --- | --- | --- | --- | --- | --- | --- | --- | --- | --- | --- | --- | --- | --- | --- | --- | --- |
|  | Model 1 | p-value | Model 2 | p-value | Model 3 | p-value | Model 4 | p-value | Model 1 | p-value | Model 2 | p-value | Model 3 | p-value | Model 4 | p-value |
| MoCA score<23 | 1.76(1.31, 2.37) | <0.0001 | 1.38(0.99, 1.90) | 0.051 | 1.55(1.06, 2.26) | 0.023 | 1.41(0.78, 2.54) | 0.24 | 2.11(1.41, 3.18) | <0.0001 | 1.55(1.01, 2.38) | 0.044 | 1.45(0.88, 2.39) | 0.13 | 0.95(0.45, 2.01) | 0.89 |
| Anxiety (GAD-7 score >8) | 1.12(0.86, 1.45) | 0.38 | 1.07(0.82, 1.39) | 0.59 | 1.06(0.77, 1.47) | 0.68 | 0.83(0.51, 1.35) | 0.46 | 1.09(0.79, 1.49) | 0.58 | 0.97(0.69, 1.35) | 0.87 | 0.87(0.57, 1.32) | 0.53 | 0.63(0.34, 1.15) | 0.14 |

Model 1: adjusted for age, sex, admission to ITU; Model2: adjusted for age, sex, index of multiple deprivation, Ethnicity, education, and admission to ITU; Model3: adjusted for Model2 + BMI,; Model4: Model3+number of long-term conditions

# Table S8- comparing the baseline characteristics and variables adjusted for using inverse probability-of-censoring weighting) between censored and uncensored participants

|  |  | **Total** | **Followed** | **Censored** | **p-value** |
| --- | --- | --- | --- | --- | --- |
|  |  | **N=2,545** | **N=1,827** | **N=718** |  |
| Sex(male) |  | 1,557 (61.2%) | 1,114 (61.0%) | 443 (61.7%) | 0.74 |
| Having Diabetes |  | 538 (21.1%) | 396 (21.7%) | 142 (19.8%) | 0.29 |
| Education | None or primary | 122 (5.4%) | 82 (4.9%) | 40 (6.5%) | 0.13 |
|  | Secondary or college (NVQ 3-4) | 1,373 (60.3%) | 1,019 (61.4%) | 354 (57.3%) |  |
|  | Degree or higher | 782 (34.3%) | 558 (33.6%) | 224 (36.2%) |  |
| Ethnicity | White | 1,893 (74.8%) | 1,400 (76.9%) | 493 (69.5%) | <0.0001 |
|  | South Asian | 198 (7.8%) | 115 (6.3%) | 83 (11.7%) |  |
|  | Black | 180 (7.1%) | 126 (6.9%) | 54 (7.6%) |  |
|  | Mixed/other | 259 (10.2%) | 180 (9.9%) | 79 (11.1%) |  |
| IMD Quintile | 1 (most deprived) | 578 (23.1%) | 409 (22.8%) | 169 (23.9%) | 0.022 |
|  | 2 | 584 (23.4%) | 397 (22.1%) | 187 (26.5%) |  |
|  | 3 | 435 (17.4%) | 335 (18.7%) | 100 (14.2%) |  |
|  | 4 | 436 (17.4%) | 309 (17.2%) | 127 (18.0%) |  |
|  | 5 (least deprived) | 468 (18.7%) | 345 (19.2%) | 123 (17.4%) |  |
| Admin duration |  | 8.0 (4.0-16.0) | 8.0 (4.0-17.0) | 7.0 (4.0-13.0) | <0.0001 |
| Refer to another Specialty | No | 1,185 (81.1%) | 822 (79.7%) | 363 (84.6%) | 0.038 |
|  | Yes | 276 (18.9%) | 210 (20.3%) | 66 (15.4%) |  |
| Muscle aches | No | 1,185 (55.9%) | 873 (57.7%) | 312 (51.6%) | 0.011 |
|  | Yes | 933 (44.1%) | 640 (42.3%) | 293 (48.4%) |  |
| No comorbidity |  | 1.0 (0.0-2.0) | 1.0 (0.0-2.0) | 0.0 (0.0-2.0) | <0.0001 |

# References:

1. *Office for National Statistics. National Statistics Postcode Lookup* December 01, 2021]; Available from: <https://geoportal.statistics.gov.uk/datasets/national-statistics-postcode-lookup-february2020>

2. Johnson, S.U., et al., *Psychometric properties of the general anxiety disorder 7-item (GAD-7) scale in a heterogeneous psychiatric sample.* Frontiers in psychology, 2019. **10**: p. 1713.

3. Yorke, J., et al., *Quantification of dyspnoea using descriptors: development and initial testing of the Dyspnoea-12.* Thorax, 2010. **65**(1): p. 21-26.

4. *FACIT.org. Functional Assessment of Chronic Illness Therapy – Fatigue: A 13-item FACIT Fatigue Scale*. March 19, 2021]; Available from: <https://www.facit.org/measures/FACIT-F>

5. Butt, Z., et al., *Measurement of fatigue in cancer, stroke, and HIV using the functional assessment of chronic illness therapy—fatigue (FACIT-F) scale.* Journal of psychosomatic research, 2013. **74**(1): p. 64-68.

6. Cleeland, C.S. *The Brief Pain Inventory User Guide*. 2009 [cited 2021 March 01]; Available from: <https://www.mdanderson.org/content/dam/mdanderson/documents/Departments-and-Divisions/Symptom-Research/BPI_UserGuide.pdf>.

7. Cleeland, C. and K. Ryan, *Pain assessment: global use of the Brief Pain Inventory.* Annals, academy of medicine, Singapore, 1994.

8. Kreutzer, J.S., J. DeLuca, and B. Caplan, *Encyclopedia of clinical neuropsychology*. 2011: Springer.

9. Guralnik, J.M., et al., *A short physical performance battery assessing lower extremity function: association with self-reported disability and prediction of mortality and nursing home admission.* Journal of gerontology, 1994. **49**(2): p. M85-M94.

10. Vasunilashorn, S., et al., *Use of the Short Physical Performance Battery Score to predict loss of ability to walk 400 meters: analysis from the InCHIANTI study.* Journals of Gerontology Series A: Biomedical Sciences and Medical Sciences, 2009. **64**(2): p. 223-229.

11. Singh, S.J., et al., *Development of a shuttle walking test of disability in patients with chronic airways obstruction.* Thorax, 1992. **47**(12): p. 1019-1024.

12. Probst, V.S., et al., *Reference values for the incremental shuttle walking test.* Respiratory medicine, 2012. **106**(2): p. 243-248.

13. Rockwood, K., et al., *A global clinical measure of fitness and frailty in elderly people.* Cmaj, 2005. **173**(5): p. 489-495.

14. *Nasreddine ZS. MoCA Montreal Cognitive Assessment Training & Certification*. March 01, 2021]; Available from: <https://www.mocatest.org/training-certification/>

15. Carson, N., L. Leach, and K.J. Murphy, *A re‐examination of Montreal Cognitive Assessment (MoCA) cutoff scores.* International journal of geriatric psychiatry, 2018. **33**(2): p. 379-388.

16. Graham, B.L., et al., *Standardization of spirometry 2019 update. An official American thoracic society and European respiratory society technical statement.* American journal of respiratory and critical care medicine, 2019. **200**(8): p. e70-e88.

17. *Acute heart failure: diagnosis and management. National Institute for Health and Care Excellence*. 2014.

18. *Chronic heart failure in adults: diagnosis and management. National Institute for Health and Care Excellence*. 2018.

19. Committee, I.E., *International Expert Committee report on the role of the A1C assay in the diagnosis of diabetes.* Diabetes care, 2009. **32**(7): p. 1327-1334.

20. Herdman, M., et al., *Development and preliminary testing of the new five-level version of EQ-5D (EQ-5D-5L).* Quality of life research, 2011. **20**(10): p. 1727-1736.

21. Gerlinger, C., et al., *Comparing the EQ-5D-5L utility index based on value sets of different countries: impact on the interpretation of clinical study results.* BMC research notes, 2019. **12**(1): p. 1-6.

22. *Creating Disability Severity Indicators Using the WG Short Set on Functioning (WG-SS) (CSPro)*. January 2021 [cited 2021 November 16]; Available from: <https://www.washingtongroup-disability.com/fileadmin/uploads/wg/WG_Document> 5H_-_Analytic_Guidelines_for_the_WG-SS Severity_Indicators_-_CSPro_.pdf
